# Traditional herbal medicine for the prevention of chemotherapy-induced peripheral neuropathy: a systematic review and meta-analysis with association rule analysis

**DOI:** 10.3389/fphar.2025.1607181

**Published:** 2025-06-30

**Authors:** Eun Hye Kim, Hayun Jin, Su Hyeon Lee, Seong Woo Yoon

**Affiliations:** ^1^ Department of Internal Medicine, College of Korean Medicine, Gachon University, Gyeonggi-Do, Republic of Korea; ^2^ Department of Korean Internal Medicine, Kyung Hee University Hospital at Gangdong, Seoul, Republic of Korea; ^3^ Department of Clinical Korean Medicine, Graduate School, Kyung Hee University, Seoul, Republic of Korea

**Keywords:** cancer, chemotherapy-induced peripheral neuropathy, traditional herbal medicine, prevent, *Astragali radix*

## Abstract

**Introduction:**

This systematic review and meta-analysis evaluated the preventive efficacy and safety of orally-administered traditional herbal medicine (THM) for the management of chemotherapy-induced peripheral neuropathy (CIPN) in patients with cancer.

**Methods:**

Randomized controlled trials (RCTs) evaluating the efficacy of orally-administered THM in the prevention of CIPN published up to 30 April 2024 were retrieved from nine databases. The primary outcome was the incidence of CIPN, and the secondary outcomes included changes in neuropathic pain intensity, nerve conduction study parameters, Karnofsky Performance Scale (KPS) scores, and the incidence of adverse events. The quality of the studies and the strength of the evidence were evaluated using the Cochrane Risk of Bias Assessment Tool and Grading of Recommendations Assessment, Development, and Evaluation (GRADE) method. Key herbal combinations were identified by conducting an association rule analysis.

**Results:**

Thirty-seven RCTs involving 2,882 patients were included. Significant differences were observed between THM and the placebo [RR 0.83, 95% CI 0.74–0.93, p < 0.05; low quality of evidence], usual care [RR 0.51, 95% CI 0.37–0.69, p < 0.05; moderate quality of evidence], and no treatment [RR 0.62, 95% CI 0.54–0.71, p < 0.05; moderate quality of evidence] in terms of in the incidence rate of CIPN. A significant reduction in the intensity of neuropathic pain [SMD -0.81, 95% CI -1.07 to −0.56, p < 0.05; high quality of evidence] and a significant improvement in KPS [MD 8.18, p < 0.05; low quality of evidence] were observed in the THM compared to no treatment. Furthermore, compared with usual care and no treatment, the use of THM yielded a significant improvement in the nerve conduction parameters with low quality of evidence. No serious adverse events were reported. The combination of Astragali Radix and Cinnamomi Ramulus as the strongest herbal combination used for the prevention of CIPN.

**Conclusion:**

THM may be a promising option for the prevention of CIPN in patients with cancer. Low certainty of evidence, and substantial heterogeneity and risk of bias can limit the strength of the conclusions. Further well-designed and rigorously reported randomized controlled trials are warranted to confirm these findings and clarify their clinical applicability.

**Systematic Review Registration:**

https://www.crd.york.ac.uk/prospero, Identifier: CRD42021270942.

## 1 Introduction

Cancer is the leading cause of death worldwide, with both incidence and mortality rates continuing to rise globally each year. Chemotherapy (CTX) is administered to 60%–75% of cancer patients as part of conventional cancer treatments, but prolonged use often leads to systemic side effects (Fitzmaurice et al., 2017). Notably, chemotherapy-induced peripheral neuropathy (CIPN) has been detected in 38%–70% of patients receiving platinum-based, taxane-based, or bortezomib chemotherapy. CIPN, characterized by the presence of symptoms such as dysesthesia, numbness, pain, cold sensitivity, sensory loss, burning sensations, and motor dysfunction including muscle cramps and reduced muscle strength (Richardson et al., 2006; Oh and Kim, 2018), is caused by damage to the peripheral motor, sensory, and autonomic nervous systems.

CIPN is particularly prevalent among patients with colorectal, gastric, breast, and hematological cancers, making it the second most frequent chemotherapy-induced side effect following myelosuppression (Windebank and Grisold, 2008). This condition arises from the damage caused by the accumulation of neurotoxic CTX drugs in the myelin sheaths of nerve cells, which destroy the peripheral nerve tissues. CIPN can persist for months or years following the completion of conventional cancer treatment, potentially leading to irreversible sequelae (Tofthagen et al., 2014). In addition to reducing the quality of life (QoL) and physical function, CIPN also delays or decreases the effectiveness of CTX. Consequently, researchers have explored various methods for its prevention and treatment. The guidelines set forth by the American Society of Clinical Oncology (ASCO) indicate that duloxetine is the only drug recommended for the treatment of patients with CIPN (Loprinizi et al., 2020). However, the drug interactions and toxicity associated with duloxetine have limited its clinical use. While anticonvulsants, opioid analgesic, and rehabilitation therapies are frequently used in practice, none have demonstrated sufficient evidence of efficacy or safety, and no pharmacological or non-pharmacological treatments have been proven to prevent CIPN (Wickham, 2007; Smith et al., 2013; Fukuda et al., 2017). The ASCO guidelines list acupuncture, compression therapy, and exercise therapy as interventions for which no recommendation can be made, primarily due to the low quality of supporting evidence. While these approaches show potential benefits, the guidelines have stated that larger sample-sized studies are needed to confirm their efficacy. Additionally, vitamin B—particularly B12—and glutathione were reported to provide no benefit, with this conclusion supported by intermediate-quality evidence. Despite this, these agents remain commonly utilized in clinical practice as part of usual care for CIPN prevention (Loprinzi et al., 2020).

The use of traditional herbal medicine (THM) as an adjunct to conventional cancer treatment has increased in recent years, with an increasing number of guidelines and studies exploring its role as a complementary and alternative medicine in standard oncological care. The combination of THM and CTX enhances the QoL and provides a synergistic effect with conventional cancer treatments (Chien, 2017; Wang et al., 2020). Notably, several studies have demonstrated the effectiveness of THM in the management of CTX-induced side effects such as anorexia, diarrhea, nausea, vomiting, and mucositis (Ohnishi and Takeda, 2015). In the context of CIPN, oral administration of THM has been shown to have therapeutic effects, as evidenced by systematic reviews and meta-analyses in certain cancers. Significant improvements in the severity of severe CIPN were observed among patients with colorectal cancer (CRC) receiving a combination of folinic acid, fluorouracil, and oxaliplatin (FOLFOX) and among those with breast cancer receiving taxane-based CTX (Noh et al., 2018; Liu et al., 2019).

Despite these findings, there remains a lack of comprehensive research and robust evidence regarding the efficacy and safety of THM in the prevention of CIPN. Therefore, this systematic review and meta-analysis of randomized controlled trials (RCTs) evaluated the efficacy and safety of orally-administered THM in the prevention of CIPN among patients with cancer. In addition, an *a priori* algorithm-based association analysis was conducted using the herbal composition data to identify key herb combinations.

## 2 Methods

### 2.1 Search strategy

This study aimed to compare the effects of orally administered THM *versus* control interventions (placebo, usual care, or no treatment) for the prevention of CIPN in cancer patients scheduled to receive CTX regimens known to commonly cause CIPN. A systematic review and meta-analysis were conducted to evaluate the preventive efficacy of THM. RCTs evaluating the efficacy of orally-administered THM in the prevention of CIPN published since the date of inception of the database to 30 April 2024 were retrieved from nine electronic databases. The databases included three English databases (PubMed, EMBASE, and the Cochrane Library), one Chinese database (Chinese National Knowledge Infrastructure Database (CNKi)), one Japanese database (Citation Information by National Institute of Information (CiNii)), and four Korean databases (Korean Medical Database (KMBASE), Korean Studies Information Service System (KISS), National Digital Science Library (NDSL), and Oriental Medicine Advanced Searching Integrated System (OASIS)). The search was conducted independently by two authors without restrictions on the date of publication or language using the following search terms: neoplasm, cancer, chemotherapy, cisplatin, taxane, neuropathy, sensory impairment, herbal medicine, traditional Chinese medicine, and decoction. The search terms were modified for each database using a highly sensitive search strategy developed by the Cochrane Collaboration. The Supplementary Material S1 presents the full details of the search strategies.

This systematic review and meta-analysis adhered to the Preferred Reporting Items for Systematic Reviews and Meta-Analysis (PRISMA) checklist (Moher et al., 2009). The study protocol was registered with the International Prospective Register of Systematic Review (PROSPERO) under the registration number CRD42021270942. Ethical approval was not required as all research materials were published studies.

### 2.2 Study selection

The selection process was independently conducted by the two authors. Any disagreements between the authors were resolved by reaching a consensus with a third researcher. The titles and abstracts of the retrieved studies were screened for relevance. Full-text articles that satisfied the following inclusion criteria were subsequently assessed: 1) RCTs (parallel and/or crossover design); 2) clinical studies focused on CIPN in patients with cancer; 3) studies with adult patients (age ≥18 years); 4) the use of orally-administered THM as an intervention for the prevention of CIPN (preventive purpose); and 5) availability of the full-text.

Studies that satisfied any of the following exclusion criteria were excluded: 1) use of THM as a part of therapeutic treatment (rather than prevention) to alleviate existing CIPN-related symptoms (therapeutic purpose); 2) studies assessing the role of non-oral administration of THM, such as intravenous, topical, washing, or fumigation; and 3) dissertations, publications limited to abstracts, protocol papers, letters, posters, and other forms of grey literature.

### 2.3 Outcome measures

The incidence of CIPN-related symptoms, including neuropathic pain, neuralgia, sensory impairment, and hand-foot pain, was defined as the primary outcome measure for evaluating the efficacy of THM in preventing the incidence of CIPN in patients with cancer. The incidence rate was defined as the proportion of patients in each group who exhibited CIPN-related symptoms following the initiation of CTX. The criteria for defining incidence were based on the definitions provided in each included study, including the Common Terminology Criteria for Adverse Events (CTCAE), and were analyzed according to the available data. The intensity of neuropathic pain, QoL scores such as the Karnofsky performance scale (KPS), and nerve conduction study (NCS) parameters for sensory and motor nerves were defined as the secondary outcome measures. Furthermore, data regarding the incidence of adverse events (AEs) were collected from studies reporting the safety of THM interventions.

### 2.4 Data extraction

Two authors independently extracted data from the included studies using a standardized data collection form. The extracted data included the following: title, the name of the first author, publication year, sample size, study design, type of cancer, CTX regimen, details of the interventions (composition, dosage, schedule, and duration), control groups (placebo, usual care, and no treatment), outcome measures, and the incidence of AEs. The outcomes were recorded for the duration corresponding to the complete administration of THM. Any disagreements between the authors were resolved by reaching a consensus through discussion with a third researcher. The corresponding authors were contacted if the studies had missing information.

### 2.5 Quality assessment

The methodological quality of the included RCTs was independently assessed by two authors using the Cochrane Risk of Bias Tool from the Cochrane Handbook version 5.2 with the following domains: random sequence generation (selection bias), allocation concealment (selection bias), blinding of participants and personnel (performance bias), blinding of outcome assessment (detection bias), incomplete outcome data (attrition bias), selective reporting (reporting bias), and other bias (unclear distribution of prognostic factors) (Higgins et al., 2017). The risk of bias in each domain was rated as “low risk,” “high risk,” and “unclear risk.” Disagreements between the authors were resolved by reaching a consensus through discussion with a third researcher.

### 2.6 Statistical analysis

The pooled data were analyzed using Review Manager (RevMan, Version 5.4, Copenhagen: The Nordic Cochrane Centre, The Cochrane Collaboration, 2014). The mean difference (MD) and risk ratios (RRs) with 95% confidence intervals (CIs) were calculated for continuous variables and dichotomous outcomes, respectively (Higgins et al., 2017). *I* ^2^ tests were conducted to assess heterogeneity. A random-effects model was applied If more than four studies were included in a comparison and significant heterogeneity was detected (with a value of *I* ^2^ ≥50%), a random-effects model was applied; otherwise, a fixed-effects model was used (Tufanaru et al., 2015). Heterogeneity across studies was considered statistically significant if the p-value from the Chi-square test was below 0.10, or if *I* ^2^ ≥50% (Higgins, 2003). Subgroup analyses were conducted to evaluate the validity of the results in the presence of heterogeneity. Potential publication bias was detected by constructing funnel plots if more than ten studies were included in the meta-analysis.

The studies were grouped according to the type of control (including placebo, usual care [vitamin B12 or daily management], and no treatment), composition of intervention (such as Huangqi-Guizahi-Wuwu Decoction [HGWD], Dang-Gui-Si-Ni-Tang [DGSNT], Gosha-jinki-gan [GJG]), and CTX regimen (such as platinum-based). The quality of evidence for each outcome, classified as “high,” “moderate,” “low,” or “very low” based on factors such as risk of bias, inconsistency, indirectness, impression, and publication bias, was assessed using the Grading of Recommendations Assessment, Development, and Evaluation (GRADE) method. Detailed criteria used for each GRADE domain (e.g., *I* ^2^ thresholds for inconsistency, confidence interval ranges for imprecision) are described in the Supplementary Material S2. High-quality evidence indicated that the true effect is close to the estimated effect. Moderate-quality evidence indicated moderate confidence in the effect estimate, i.e., the true effect is likely to be close to the estimate of the effect; however, there is a possibility that it is substantially different. Low-quality evidence indicated limited confidence in the effect estimate, i.e., the true effect may differ substantially from the effect estimate. Very low-quality evidence indicated very little confidence in the effect estimate, i.e., the true effect is likely to differ substantially from the estimate of effect (GRADE Working Group, 2004).

In conducting this meta-analysis, we also assessed the assumptions of transitivity and consistency to ensure the validity of indirect comparisons and pooled estimates. Transitivity was evaluated by conducting subgroup analyses based on cancer type, CTX regimen, and outcome measurement methods, assuming these factors could influence treatment effects across studies. Consistency was assessed by examining the direction and magnitude of effect sizes across studies and by using *I* ^2^ statistics, which directly informed the GRADE assessment. To further explore the robustness of the findings, sensitivity analyses were conducted by excluding studies with a high risk of bias.

The key herb combinations in the THM compositions used in the included studies were identified by conducting an *a priori* algorithm-based association analysis. The frequency of individual herbs was assessed to identify the most frequently used combinations. *A priori* association rule analysis was performed using Statistical Package for the Social Sciences (SPSS) Statistics (version 26.0), with the findings being visualized by generating plots (Agrawal et al., 1993). The primary metrics used to evaluate associations were support, confidence, and lift. 1) The metric “Support” measures the usefulness of an association rule, representing the proportion of prescriptions containing a specific herb combination relative to the total number of THM prescriptions. 2) The metric “Confidence” indicates the likelihood of the consequent herb set being included when a specific antecedent herb set is present in a THM prescription. 3) The metric “Lift” adjusts for the fact that it is not known whether the confidence is useful or a random result. For instance, the confidence of herbs A and B was divided by the confidence under the independent assumption that A does not affect B. When the confidence is approximately 1, herbs A and B are considered unrelated. Conversely, a higher lift value indicated a stronger association (Jo and Lee, 2021). The association rules were identified using minimum thresholds of 15% support and 85% confidence in the present study. The analysis focused on the identification of the core herb combinations with the most distinct associations. The constituent herbs of these combinations were examined further.

## 3 Results

### 3.1 Study selection

**FIGURE 1:**
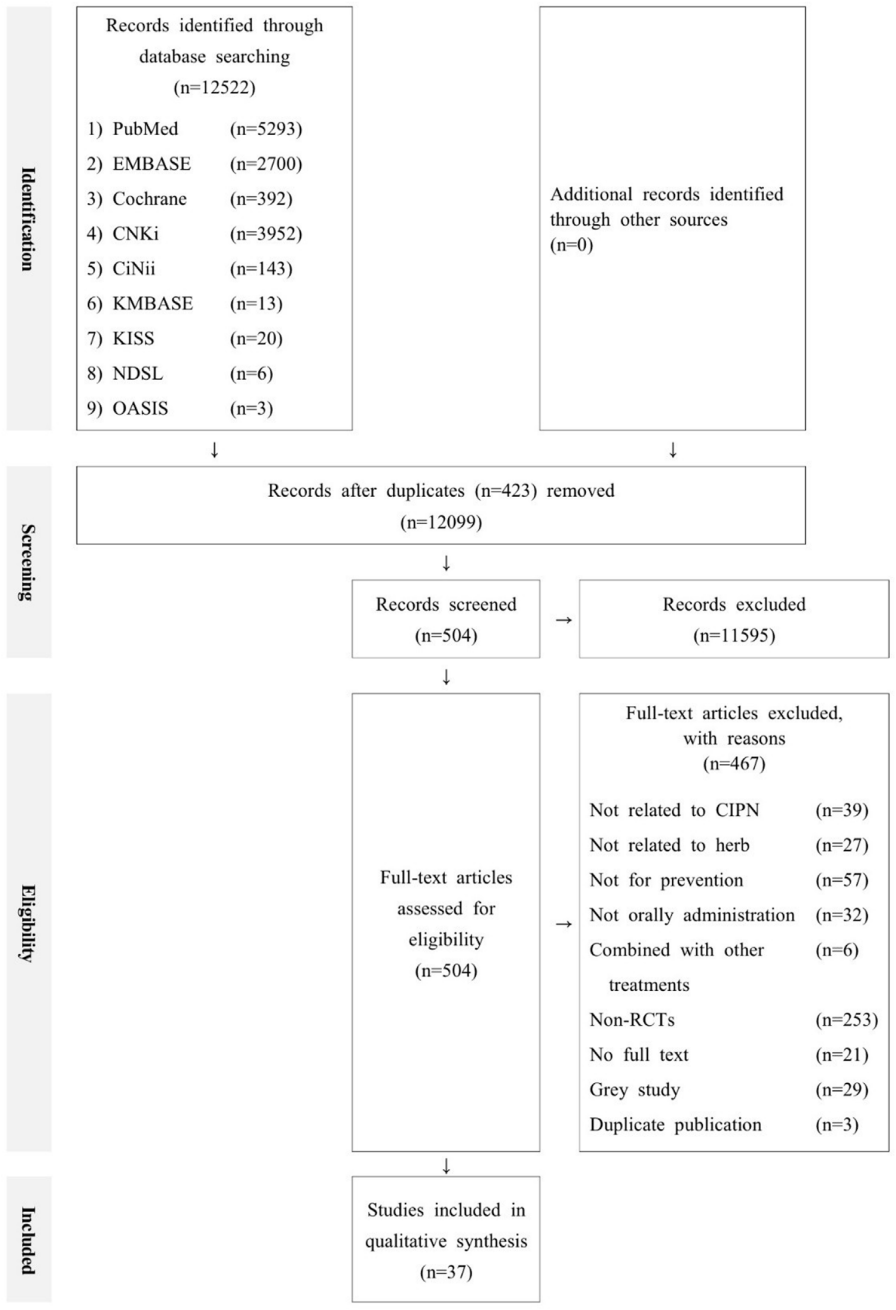
PRISMA flowchart of study selection.

A total of 12,522 potentially relevant studies were identified across nine databases using the search strategy. Among them, 12,099 records were retained for screening following the exclusion of 423 duplicate records. Screening of the titles and abstracts led to the exclusion of 11,595 articles that met at least one of the exclusion criteria. Full-text assessments of the remaining 504 studies led to the exclusion of 468 articles for the following reasons: unrelated to CIPN (n = 39); unrelated to herbal medicine (n = 27); not for preventive purpose (n = 57); non-oral administration (n = 32); combined with other interventions (n = 6); not RCTs (n = 253); unavailability of the full text (n = 21); grey literature (n = 29), and duplicated publications (n = 3). Thus, 37 studies that met the inclusion criteria were included in this systematic review and subsequent meta-analysis. Figure 1 presents a detailed flowchart of the study selection process.

### 3.2 Study characteristics

Table 1 summarizes the characteristics of the included studies. The 37 RCTs included in the present study were published between 2006 and 2024. Most studies were conducted in China (n = 32) (Li et al., 2006; Jia et al., 2008; Lin et al., 2009; Xu, 2010; Liu et al., 2011; Bo and Wenling, 2012; Tao et al., 2012; Wu et al., 2012; Liu et al., 2013; Ding et al., 2014; Yu et al., 2014; Wu et al., 2015; Zhang et al., 2015; Bai and Shi, 2016; Tong, 2016; Wang Q., et al., 2016; Xu, 2016; Cheng et al., 2017; Wang, 2017; Xu et al., 2017; Chen et al., 2018; Fan et al., 2018; Ren and Wang, 2018; Su and Huang, 2018; Zhang W., 2018; Zhang Y., 2018; Xi et al., 2019; Liu et al., 2020; Lyu et al., 2021; Ho et al., 2022; Zu et al., 2023; Yu et al., 2024). The remaining studies were conducted in Japan (n = 5) (Nishioka et al., 2011; Abe et al., 2013; Oki et al., 2015; Zhang Y., 2018; Motoo et al., 2020). The sample sizes ranged from 40 to 182 participants. Seventeen RCTs included patients with gastrointestinal cancer, particularly those with CRC (Lin et al., 2009; Xu and Ding, 2010; Nishioka et al., 2011; Wu et al., 2012; Liu et al., 2013; Yu et al., 2014; Oki et al., 2015; Bai and Shi, 2016; Cheng et al., 2017; Wang, 2017; Chen et al., 2018; Xi et al., 2019; Motoo et al., 2020; Lyu et al., 2021; Ho et al., 2022; Zu et al., 2023; Yu et al., 2024). Two RCTs involved patients with ovarian cancer (Yu et al., 2014; Xu, 2016), four involved patients with lung cancer (Lin et al., 2009; Xu and Ding, 2010; Tao et al., 2012; Yu et al., 2014), one involved patients with breast cancer (Abe et al., 2013), and one involved patients with multiple myeloma (Liu et al., 2020). Sixteen studies did not specify the type of cancer assessed (Li et al., 2006; Jia et al., 2008; Liu et al., 2011; Bo and Wenling, 2012; Kono et al., 2013; Ding et al., 2014; Wu et al., 2015; Zhang et al., 2015; Tong, 2016; Wang Q., et al., 2016; Xu et al., 2017; Fan et al., 2018; Ren and Wang, 2018; Su and Huang, 2018; Zhang W., 2018; Zhang Y., 2018). The cancer stage, which was stage III CRC, was reported in only one study (Motoo et al., 2020).

**TABLE 1:** Basic characteristics of included studies.

Study ID (Year)	Cancer type	Regimen of CTX	N (I/C)	THM	Control	Duration	Outcome (Tool)	AEs (THM vs. control; %)
Oki et al. (2015)	CRC	mFOLFOX6	89/93	GJG (7.5g, t.i.d.)	Placebo	12 cycles of CTX (2 weeks as one cycle)	Incidence rate of CIPN (CTCAE)	Anorexia (68.9 vs. 73.1), fatigue (65.6 vs. 66.7), nausea (72.2 vs. 76.3), vomiting (25.6 vs. 33.3), diarrhea (35.6 vs. 30.1), allergic reaction (16.7 vs. 18.3), chromatosis (21.1 vs. 18.3), anemia (60.0 vs. 55.9), leucopenia (61.1 vs. 63.4), neutropenia (70.0 vs. 75.3), thrombocytopenia (61.1 vs. 50.5) (*p* > 0.05)
Liu et al. (2013)	CRC	Oxaliplatin	60/60	Tong-luo fang (200mL, b.i.d.)	Placebo	2 cycles of CTX (2 weeks as one cycle)	Incidence rate of CIPN (CTCAE)	Anemia (11.7 vs. 13.3), neutropenia grade 1–2/3–4 (23.3 vs. 21.7/11.7 vs. 10.0), thrombocytopenia (16.7 vs. 15.0), nausea (30.0 vs. 33.3), vomiting (23.3 vs. 26.7), diarrhea grade 1–2/3–4 (20.0 vs. 21.7/1.7 vs. 5.0), stomatitis (20.0 vs18.3) (*p* > 0.05)
Kono et al. (2013)	NR	FOLFOX4, mFOLFOX6	44/45	GJG (7.5g, t.i.d.)	Placebo	8 weeks	Incidence rate of CIPN (CTCAE)	Vomiting (9 vs. 29; *p* = 0.029), AST elevation (30 vs. 31; *p* = 0.052), ALT elevation (23 vs. 42; *p* = 0.0070)
Cheng et al. (2017)	CRC	FOLFOX	36/36	HGWD (b.i.d.)	Placebo	Four cycles of CTX (2 months)	Incidence rate of CIPN (Levi’s scale)	Vomiting (8.33 vs. 5.56), nausea (25 vs. 19.44), constipation (8.33 vs. 11.11), anorexia (27.78 vs. 41.67), and insomnia (2.78 vs. 0) (*p* = 0.6407)
Zhang Y. (2018)	NR	Oxaliplatin	40/40	Decoction for individual research (daily)	Usual care (avoiding cold/heat sensation)	28 days	Incidence rate of CIPN (study-specific; 0–4 Grade)	NR
Ren and Wang (2018)	NR	Paclitaxel	30/30	HGWD (b.i.d.)	Vitamin B 12 (p.o., t.i.d.)	21 days	Incidence rate of CIPN (Levi’s scale)	NR
Yu et al. (2014)	Ovary, Esophagus, NSCLC	Paclitaxel plus cisplatin	25/25	HGWD (b.i.d.)	Vitamin B 12 (p.o., t.i.d.)	14 days	Incidence rate of CIPN (CTCAE) NCS	NR
Xi et al. (2019)	CRC	FOLFOX	75/75	Decoction for strengthen the spleen (b.i.d.)	Usual care (details NR)	24 weeks	Incidence rate of CIPN (Levi’s scale)	NR
Xu et al. (2017)	NR	FOLFOX	34/34	HGWD (b.i.d.)	Vitamin B 12 (p.o., t.i.d.)	Four cycles of CTX (2 weeks as one cycle)	Incidence rate of CIPN (Levi’s scale)	NR
Xu (2016)	Ovary cancer	Paclitaxel plus cisplatin	38/38	HGWD (b.i.d.)	Vitamin B 12 (p.o., t.i.d.)	Six cycles of CTX (3 weeks as one cycle)	Incidence rate of CIPN (CTCAE)	NR
Liu et al. (2011)	NR	Oxaliplatin	28/29	HGWD (t.i.d.)	Vitamin B 12 (p.o., t.i.d.)	42 days	Incidence rate of CIPN (Levi’s scale), NCS	NR
Abe et al. (2013)	BC	Docetaxel	33/27	GJG (7.5g, b.i.d. Or t.i.d.)	Vitamin B 12 (p.o., t.i.d.)	Six cycles of CTX (3 weeks as one cycle)	Incidence rate of CIPN (CTCAE)	Leucopenia (55 vs. 56), neutropenia (55 vs. 59, febrile neutropenia (3 vs. 0), fatigue (45 vs. 51), nausea/vomiting (36 vs. 33), anorexia (33 vs. 48), stomatitis (27 vs. 30), diarrhea (21 vs. 19), rash/eczema (18 vs. 19), AST/ALT elevation (9 vs. 11), nail change (27 vs. 26), peripheral edema (18 vs. 26) (*p* > 0.05)
Xu and Ding (2010)	Lung cancer, CRC	Oxaliplatin	32/22	Yiqi Huoxue Decoction (b.i.d.)	No treatment	Six cycles of CTX (6 months)	Incidence rate of CIPN (Levi’s scale)	NR
Ding et al. (2014)	NR	Oxaliplatin	24/24	DGSNT (b.i.d.)	No treatment	84 days	Incidence rate of CIPN (Levi’s scale), pain intensity score	NR
Zhang Y. (2018)	NR	FOLFOX4	30/30	Yanghe Decoction (b.i.d.)	No treatment	Four cycles of CTX (3 weeks as one cycle)	Incidence rate of CIPN (Levi’s scale)	NR
Zhang et al. (2015)	NR	Oxaliplatin	30/30	Bazhen Decoction (NR)	No treatment	Two cycles of CTX (3 weeks as one cycle)	Incidence rate of CIPN (Levi’s scale), KPS	NR
Chen et al. (2018)	Gastric, rectal cancer	mFOLFOX4	30/31	HGWD (t.i.d.)	No treatment	NR	Incidence rate of CIPN (CTCAE)	NR
Wang (2017)	Rectal cancer	FOLFOX4	50/50	Lizhong Decoction (b.i.d.)	No treatment	21 days	Incidence rate of CIPN (CTCAE)	NR
Wu et al. (2015)	NR	Paclitaxel or vincristine	30/30	HGWD (b.i.d.)	No treatment	Two cycles of CTX (3 weeks as one cycle)	Incidence rate of CIPN (Levi’s scale)	NR
Wu et al. (2012)	GI cancer	Oxaliplatin	20/20	Bu-yang-huan-wu-tang (b.i.d.)	No treatment	Eight cycles of CTX	Incidence rate of CIPN (Levi’s scale), NCS	NR
Wu et al. (2012)	GI cancer	Oxaliplatin	20/20	Sijunzi Decoction plus Shingi-whan (b.i.d.)	No treatment	Eight cycles of CTX	Incidence rate of CIPN (Levi’s scale), NCS	NR
Su and Huang (2018)	NR	FOLFOX	25/25	HGWD (b.i.d.)	No treatment	Six cycles of CTX (2 weeks as one cycle)	Incidence rate of CIPN (Levi’s scale), NCS	NR
Fan et al. (2018)	NR	XELOX	31/29	Jianpijiedu Decoction (NR)	No treatment	Eight cycles of CTX (3 weeks as one cycle)	Incidence rate of CIPN (CTCAE)	NR
Bai and Shi (2016)	CRC	FOLFOX or XELOX	21/30	Tongmai Sini Decoction (b.i.d.)	No treatment	Four cycles of CTX (3 weeks as one cycle)	Incidence rate of CIPN (Levi’s scale)	NR
Lin et al. (2009)	Lung, colorectal cancer	Oxaliplatin	32/22	Yiqi Huoxue Decoction (b.i.d.)	No treatment	6 months	Incidence rate of CIPN (Levi’s scale)	NR
Liu et al. (2011)	NR	Oxaliplatin	28/28	HGWD (b.i.d.)	No treatment	42 days	Incidence rate of CIPN (Levi’s scale)	NR
Tong (2016)	NR	XELOX	54/51	Decoction for individual research (b.i.d.)	No treatment	84 days	Incidence rate of CIPN (Levi’s scale), pain intensity score	NR
Wang Q. et al. (2016)	NR	FOLFOX or XELOX	30/30	DGSNT (b.i.d.)	No treatment	Two cycles of CTX (3 weeks as one cycle)	Incidence rate of CIPN (CTCAE)	NR
Nishioka et al. (2011)	Colon cancer	FOLFOX	22/23	GJG (7.5 g)	No treatment	10 cycles of CTX (2 weeks as one cycle)	Incidence rate of CIPN (CTCAE)	Neutropenia (14 vs. 4), anorexia (0 vs. 4), nausea (18 vs. 9), vomiting (5 vs. 4), diarrhea (9 vs. 17), mucositis (9 vs. 9), and all grade 3 toxicity (36 vs. 35) (*p* > 0.05)
Motoo et al. (2020)	CRC stage 3	CapeOX	20/20	Ninjin’yoeito (9 g)	No treatment	Eight cycles of CTX (3 weeks as one cycle)	Incidence rate of CIPN (CTCAE)	Anorexia (10 vs. 35), nausea/vomiting (0 vs. 15), neutropenia (15 vs. 25), thrombocytopenia (20 vs. 5), general malaise (5 vs. 2), insomnia (0 vs. 5)
Li and Weng (2017)	NR	Oxaliplatin	31/31	HGWD (b.i.d.)	No treatment	Two cycles of CTX (3 weeks as one cycle)	Incidence rate of CIPN (CTCAE)	NR
Bo and Wenling (2012)	NR	FOLFOX4	41/44	Decoction for strengthen the spleen (b.i.d.)	No treatment	6 months	Incidence rate of CIPN (study-specific, 0–3 Grade)	Leukopenia (22 vs. 23), thrombocytopenia (10 vs. 9), erythropenia (15 vs. 16), vomiting (41 vs. 86; *p* < 0.05), diarrhea (7 vs. 20; *p* < 0.05)
Jia et al. (2008)	NR	Oxaliplatin	40/40	Bu-yang-huan-wu-tang (NR)	No treatment	Three cycles of CTX (3 weeks as one cycle)	Incidence rate of CIPN (Levi’s scale)	NR
Liu et al. (2020)	Multiple myeloma	Bortezomib	40/42	Decoction for individual research (b.i.d.)	No treatment	Six cycles of CTX (3 weeks as one cycle)	Incidence rate of CIPN (CTCAE)	NR
Tao et al. (2012)	NSCLC	NR	46/45	Decoction for individual research (b.i.d.)	No treatment	Four cycles of CTX (4 weeks as one cycle)	Incidence rate of CIPN (CTCAE)	NR
Zu et al. (2023)	Sigmoid colon cancer	XELOX	44/44	Decoction for individual research (b.i.d.)	No treatment	Two cycles of CTX (3 weeks as one cycle)	Incidence rate of CIPN (CTCAE) KPS	NR
Ho et al. (2022)	CRC	FOLFOX	60/60	Tong-luo Decoction (NR)	No treatment	Four cycles of CTX (3 weeks as one cycle)	Incidence rate of CIPN (CTCAE), KPS	NR
Yu et al. (2014)	Gastric cancer	Oxaliplatin plus Capecitabine/TS-1	30/30	HGWD (b.i.d.)	No treatment	Four cycles of CTX (2 weeks as one cycle)	Incidence rate of CIPN (Levi’s scale)	NR
Lyu et al. (2021)	GI cancer	mFOLFOX6	53/53	Decoction for individual research (NR)	No treatment	Six cycles of CTX (27 weeks)	Incidence rate of CIPN (CTCAE), pain intensity score	NR

Abbreviations: CTX, chemotherapy; N, number; I, intervention; C, control; CRC, colorectal cancer; GJG, Gosha-jinki-gan; g, gram; t. i.d, ter in die; CIPN, chemotherapy-induced peripheral neuropathy; CTCAE, common terminology criteria for adverse events; AST, aspartate aminotransferase; ALT, alanine aminotransferase; NR, not reported; mL, milliliter; b. i.d, bis in die; HGWD, Huangqi-Guizahi-Wuwu Decoction; p. o., per os; NSCLC, non-small cell lung cancer; BC, breast cancer; DGSNT, Dang-Gui-Si-Ni-Tang; GI, gastrointestinal; i. v., intra-venous; NCS, nerve conduction study; KPS, karnofsky performance scale.

The most common regimen of CTX in the included studies was platinum-based, used in 32 RCTs (Li et al., 2006; Jia et al., 2008; Lin et al., 2009; Xu and Ding, 2010; Liu et al., 2011; Nishioka et al., 2011; Bo and Wenling, 2012; Wu et al., 2012; Kono et al., 2013; Liu et al., 2013; Ding et al., 2014; Yu et al., 2014; Oki et al., 2015; Zhang et al., 2015; Bai and Shi, 2016; Tong, 2016; Wang Q. et al., 2016; Xu, 2016; Cheng et al., 2017; Wang, 2017; Xu et al., 2017; Chen et al., 2018; Fan et al., 2018; Su and Huang, 2018; Zhang W., 2018; Zhang Y., 2018; Xi et al., 2019; Motoo et al., 2020; Lyu et al., 2021; Ho et al., 2022; Zu et al., 2023; Yu et al., 2024). Taxane-based regimens were used in five RCTs (Abe et al., 2013; Yu et al., 2014; Wu et al., 2015; Xu, 2016; Ren and Wang, 2018). Bortezomib was used in one RCT (Liu et al., 2020). The CTX regimen used was not mentioned in one RCT (Tao et al., 2012). Various compositions of THM decoctions were used as interventions in the included RCTs. The HGWD, which was prescribed in 11 RCTs was the most frequently used THM decoction (Li et al., 2006; Liu et al., 2011; Yu et al., 2014; Wu et al., 2015; Xu, 2016; Cheng et al., 2017; Xu et al., 2017; Chen et al., 2018; Ren and Wang, 2018; Su and Huang, 2018; Zhang Y., 2018; Yu et al., 2024). GJG, prescribed in four studies, was the second most frequently used decoction (Nishioka et al., 2011; Abe et al., 2013; Oki et al., 2015; Zhang Y., 2018). DGSNT was prescribed in two studies (Ding et al., 2014, Wang Q., et al., 2016). Similarly, Bu-yang-huan-wu-tang was prescribed in two studies (Jia et al., 2008; Wu et al., 2012). A combination of Sijunzi decoction and Shingi-whan was prescribed in one study (Wu et al., 2012). Decoctions with personalized compositions were used in the remaining 18 RCTs (Lin et al., 2009; Xu and Ding, 2010; Bo and Wenling, 2012; Tao et al., 2012; Liu et al., 2013; Zhang et al., 2015; Bai and Shi, 2016; Tong, 2016; Wang, 2017; Fan et al., 2018; Zhang W., 2018; Zhang Y., 2018; Xi et al., 2019; Liu et al., 2020; Motoo et al., 2020; Lyu et al., 2021; Ho et al., 2022; Zu et al., 2023). *Astragalus mongholicus Bunge [*Fabaceae*; Astragali Radix]* was the most frequently used single herb. The Supplementary Material S2 provides further details regarding the THM prescriptions. Each decoction was prepared according to the composition described in the Supplementary Material S3, with the dosage of each herb adjusted proportionally to ensure that the most dominant herb did not exceed 40 g. All decoctions were administered orally in the form of aqueous extracts. Each dose of the dried herbs was decocted in water two or three times to yield 100–150 mL per decoction, and the total volume (up to 500 mL) was combined and divided into two or three portions for administration two or three times daily.

The control groups received a placebo (Kono et al., 2013; Liu et al., 2013; Oki et al., 2015; Cheng et al., 2017), usual care including avoiding cold/heat sensation (Zhang Y., 2018) and vitamin B12 (Liu et al., 2011; Abe et al., 2013; Yu et al., 2014; Xu, 2016; Xu et al., 2017; Ren and Wang, 2018), or no treatment (Li et al., 2006; Jia et al., 2008; Lin et al., 2009; Xu and Ding, 2010; Nishioka et al., 2011; Bo and Wenling, 2012; Tao et al., 2012; Wu et al., 2012; Ding et al., 2014; Wu et al., 2015; Zhang et al., 2015; Bai and Shi, 2016; Tong, 2016; Wang Q. et al., 2016; Wang, 2017; Chen et al., 2018; Fan et al., 2018; Su and Huang, 2018; Zhang Y., 2018; Motoo et al., 2020; Liu et al., 2020; Lyu et al., 2021; Ho et al., 2022; Zu et al., 2023; Yu et al., 2024). No details were provided in one RCT (Xi et al., 2019). THM and control interventions were initiated concurrently with the CTX in all studies. The duration of CTX and interventions ranged from 4 to 27 weeks, with only one study (Chen et al., 2018) reporting no specific data regarding the duration of use.

Four studies compared the efficacy of orally-administered THM with that of a placebo (Kono et al., 2013; Liu et al., 2013; Oki et al., 2015; Cheng et al., 2017), with treatment durations of 4, 8 (2 months), and 24 weeks. Three studies included patients with CRC (Liu et al., 2013; Oki et al., 2015; Cheng et al., 2017). The type of cancer was not specified in one study (Kono et al., 2013). CIPN was induced by platinum-based CTX, such as modified FOLFOX (including oxaliplatin), in all studies.

Eight studies compared the efficacy of orally-administered THM with that of usual care (Liu et al., 2011; Abe et al., 2013; Yu et al., 2014; Xu, 2016; Xu et al., 2017; Ren and Wang, 2018; Zhang Y., 2018; Xi et al., 2019), with the treatment duration ranging from 2 to 18 weeks. One study included various types of cancer (Yu et al., 2014), three studies focused on CRC (XI et al., 2019), ovarian (Xu, 2016), and breast cancer (Abe et al., 2013), respectively, while the remaining four studies did not mention the type of cancer in the enrolled participants (Zhang Y., 2018; Ren and Wang, 2018; XU et al., 2017; Liu et al., 2011). CIPN was induced by oxaliplatin as a part of multiple regimens in four studies (Liu et al., 2011; Xu et al., 2017; Zhang Y., 2018; Xi et al., 2019), paclitaxel in one study (Ren and Wang, 2018), docetaxel in one study (Abe et al., 2013), and a combination of paclitaxel and cisplatin in two studies (Yu et al., 2014; Xu, 2016). Most studies have used vitamin B12 as a part of usual care (Liu et al., 2011; Abe et al., 2013; Yu et al., 2014; Xu, 2016; Xu et al., 2017; Ren and Wang, 2018; XI et al., 2019).

Twenty-six studies compared orally-administered THM with that of no treatment (Li et al., 2006; Jia et al., 2008; Lin et al., 2009; Xu and Ding, 2010; Liu et al., 2011; Nishioka et al., 2011; Bo and Wenling, 2012; Tao et al., 2012; Wu et al., 2012; Ding et al., 2014; Wu et al., 2015; Zhang et al., 2015; Bai and Shi, 2016; Tong, 2016; Wang Q. et al., 2016; Wang, 2017; Chen et al., 2018; Fan et al., 2018; Su and Huang, 2018; Zhang Y., 2018; Liu et al., 2020; Motoo et al., 2020; Lyu et al., 2021; Ho et al., 2022; Zu et al., 2023; Yu et al., 2024), with the treatment duration ranging from 6 to 27 weeks.

Two studies included various types of cancer (Lin et al., 2009; Xu and Ding, 2010), ten studies focused on GI cancers (Nishioka et al., 2011; Wu et al., 2012; Bai and Shi, 2016; Wang, 2017; Chen et al., 2018; Motoo et al., 2020; Lyu et al., 2021; Ho et al., 2022; Zu et al., 2023; Yu et al., 2024), two studies enrolled patients with multiple myeloma (MM) (LIU et al., 2020), and lung cancer (TAO et al., 2012), respectively, while the remaining twelve studies did not mention the type of cancer in the participants (Li et al., 2006; Jia et al., 2008; Liu et al., 2011; Bo and Wenling, 2012; Ding et al., 2014; Wu et al., 2015; Zhang et al., 2015; Tong, 2016; Wang Q. et al., 2016; Fan et al., 2018; Su and Huang, 2018; Zhang Y., 2018). CIPN was induced by oxaliplatin as a part of multiple CTX regimens in 23 studies (Li et al., 2006; Jia et al., 2008; Lin et al., 2009; Xu and Ding, 2010; Nishioka et al., 2011; Liu et al., 2011; Bo and Wenling, 2012; Wu et al., 2012; Ding et al., 2014; Zhang et al., 2015; Bai and Shi, 2016; Tong, 2016; Wang Q. et al., 2016; Wang, 2017; Chen et al., 2018; Fan et al., 2018; Su and Huang, 2018; Zhang Y., 2018; Motoo et al., 2020; Lyu et al., 2021; Ho et al., 2022; Zu et al., 2023; Yu et al., 2024). CIPN was induced by paclitaxel in one study (Wu et al., 2015). Bortezomib induced CIPN in patients with MM in one study (Liu et al., 2020). The regimen was not specified in the remaining one study (Tao et al., 2012).

All included studies reported the incidence rate of CIPN, which is the primary outcome of this study. As for secondary outcome, three RCTs reported neuropathic pain intensity based on symptom questionnaires (Ding et al., 2014; Tong, 2016; Lyu et al., 2021). QoL was assessed using the KPS in three studies (Zhang et al., 2015; Ho et al., 2022; Zu et al., 2023). The NCS parameters for the sensory and motor nerves were reported in four RCTs (Liu et al., 2011; Wu et al., 2012; Yu et al., 2014; Su and Huang, 2018).

### 3.3 Risk of bias in the included studies

**FIGURE 2:**
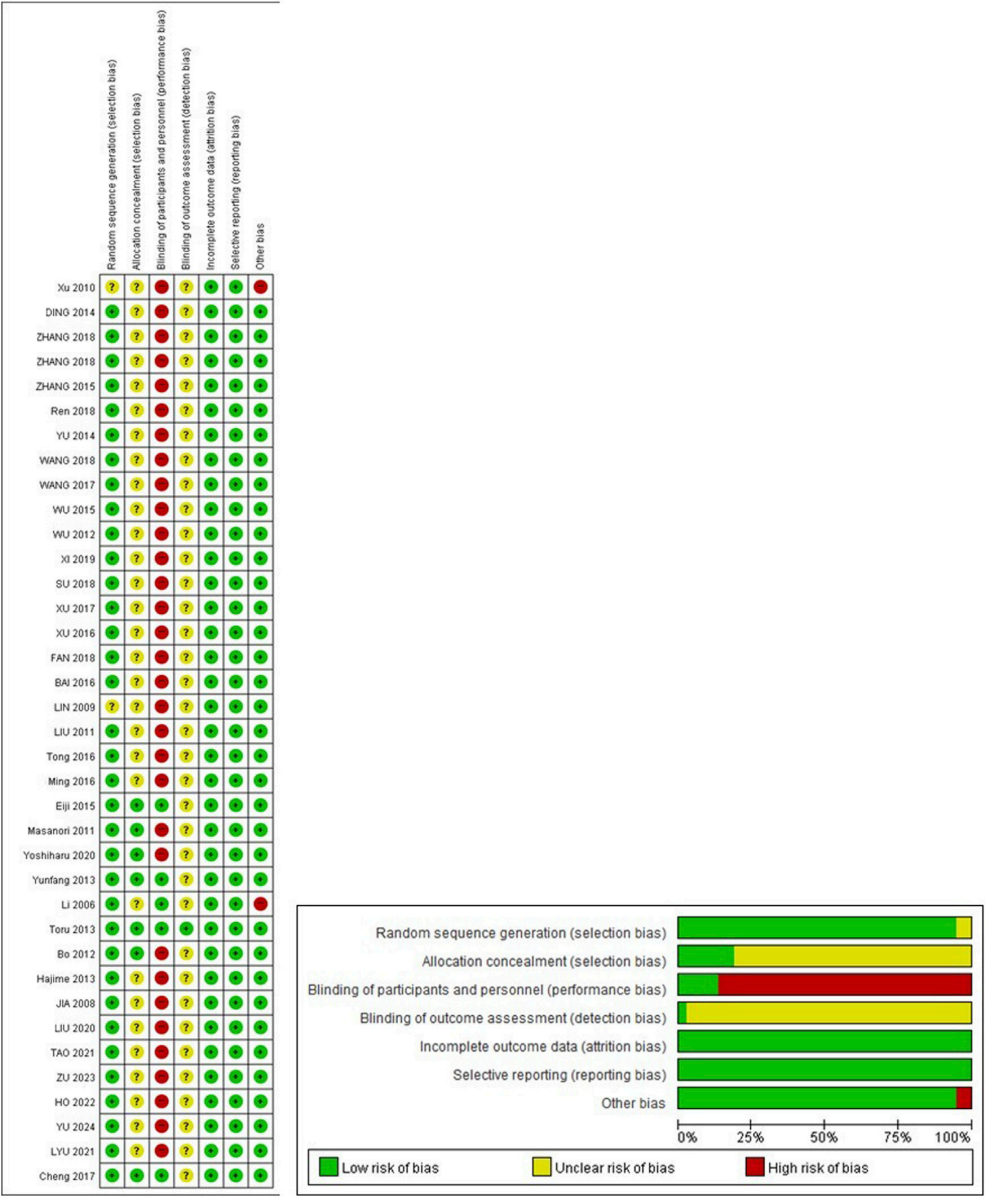
Risk of bias. +, low risk of bias; ? unclear of bias; -, high risk of bias.

The risk of bias in the included studies is shown in Figure 2. Random sequence generation was adequately described in most studies; however, an unclear selection bias was observed in two studies (Lin et al., 2009; Xu and Ding, 2010). Although these studies were reported as RCTs, there were no specific mention of the method used for randomization. In terms of allocation concealment, seven studies reported the detailed allocation procedure (Nishioka et al., 2011; Bo and Wenling, 2012; Kono et al., 2013; Liu et al., 2013; Oki et al., 2015; Cheng et al., 2017; Motoo et al., 2020). The remaining 30 studies were assessed as having an unclear risk of bias. Blinding of participants and personnel was conducted in only five studies-four of which used placebo (Kono et al., 2013; Liu et al., 2013; Oki et al., 2015; Cheng et al., 2017), and one employed a cross-over study design (Li et al., 2006). Blinding of outcome assessment was reported in only one study (Kono et al., 2013), whereas the other studies laced sufficient detail and were therefore assessed as unclear. In terms of incomplete outcome data and selective reporting bias, all studies had a low risk of bias. Two studies exhibited other biases due to baseline differences between the experimental and control groups (Xu and Ding, 2010) and crossover designs (Li et al., 2006). The remaining 35 studies had a low risk of bias.

### 3.4 Incidence rate of CIPN

All included studies reported the incidence rate of CIPN. Among the evaluation tools used to assess the incidence of CIPN, the Common Terminology Criteria for Adverse Events (CTCAE) was the most frequently applied, used in 18 studies (Table 1). The second most commonly used tool was Levi’s scale, which was employed in 17 studies. Levi’s scale classifies the severity of neuropathy from grade 0 to 4 as follows: Grade 0 indicates no clinical symptoms; Grade 1, the presence of sensory abnormality or hypoesthesia that resolves completely within 1 week; Grade 2, resolution within 21 days; Grade 3, incomplete resolution within 21 days; and Grade 4, the presence of functional impairment. Two studies used study-specific tools, which assessed CIPN by grading symptoms from grade 0 to 3 or 0 to 4 based on both the incidence of CIPN-related symptoms and their impact on daily life activities.

#### 3.4.1 Incidence rate of CIPN: comparison with placebo

**FIGURE 3:**
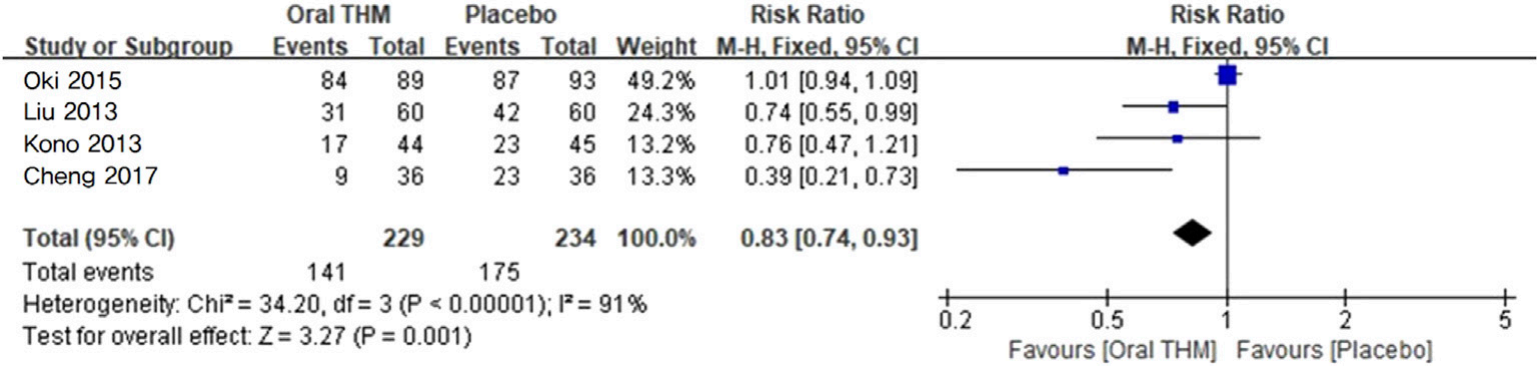
Forest plot of the incidence rate of CIPN: THM *versus* placebo. THM, traditional herbal medicine; CIPN, chemotherapy-induced peripheral neuropathy.

Four RCTs with 463 participants that reported the incidence rate of CIPN in comparison with a placebo to assess preventive efficacy were included in the meta-analysis, as shown in Figure 3 (Kono et al., 2013; Liu et al., 2013; Oki et al., 2015; Cheng et al., 2017). Significant differences were observed between THM and placebo (RR 0.83, 95% CI 0.74–0.93; p < 0.05), with a high level of heterogeneity (*I* ^2^ = 91%). This heterogeneity appears to be influenced by variations across individual studies, such as Oki et al. (2015), which reported a fixed-effect model risk ratio of 1.01 (95% CI 0.94–1.09), and Kono et al. (2013), which reported 0.76 (95% CI 0.47–1.21). Considering this heterogeneity, the GRADE assessment indicated that the quality of evidence for the incidence rate of CIPN with THM compared to placebo was low (Table 2).

**TABLE 2:** Summary of findings.

Comparison of traditional herbal medicine with placebo, usual care, or no treatment for the prevention of chemotherapy-induced peripheral neuropathy in patients with cancer						
Patient or population: Patients with cancer scheduled to undergo chemotherapy that induces peripheral neuropathy as a side effect Intervention: Traditional herbal medicine Comparison: Placebo						

Outcomes	Anticipated absolute effects (95% CI)		Relative effect (95% CI)	No. Of participants (studies)	Certainty of the evidence (GRADE)	Comments
	Risk with THM	Risk with placebo				
Incidence rate of CIPN	616 per 1,000 (194–52 fewer)	748 per 1,000	RR 0.83 (0.74–0.93)	463 (4 RCTs)	⊕⊕○○ Low	-
Intervention: traditional herbal medicine Comparison: usual care						

Outcomes	Anticipated absolute effects (95% CI)		Relative effect (95% CI)	No. Of participants (studies)	Certainty of the evidence (GRADE)	Comments
	Risk with THM	Risk with Usual care				
Incidence rate of CIPN	353 per 1,000 (336–222 fewer)	634 per 1,000	RR 0.51 (0.37–0.69)	601 (8 RCTs)	⊕⊕⊕○ Moderate	—
Nerve conduction study	MD 3.94 higher (2.78–5.11 higher)	—	—	212 (2 RCTs)	⊕⊕○○ Low	—
Intervention: traditional herbal medicine Comparison: no treatment						

Outcomes	Anticipated absolute effects (95% CI)		Relative effect (95% CI)	No. Of participants (studies)	Certainty of the evidence (GRADE)	Comments
	Risk with THM	Risk with No Tx				
Incidence rate of CIPN	415 per 1,000 (312–197 fewer)	679 per 1,000	RR 0.62 (0.54–0.71)	1,818 (26 RCTs)	⊕⊕⊕○ Moderate	—
Neuropathic pain intensity	SMD 0.81 lower (1.07–0.56 lower)	—	—	259 (3 RCTs)	⊕⊕⊕⊕ High	—
Karnofsky performance scale	MD 8.18 higher (5.89–10.47 higher)	—	—	268 (3 RCTs)	⊕⊕○○ Low	—
Nerve conduction study	MD 1.90 higher (1.08–2.72 higher)	—	—	292 (3 RCTs)	⊕⊕○○ Low	—

Abbreviations: CI, confidence interval; THM, traditional herbal medicine; CIPN, chemotherapy-induced peripheral neuropathy; MD, mean difference; RR, risk ratio; Tx, treatment; RCTs, randomized controlled trials; GRADE, grading of recommendations assessment, Development, and Evaluation.

#### 3.4.2 Incidence rate of CIPN: comparison with usual care

**FIGURE 4:**
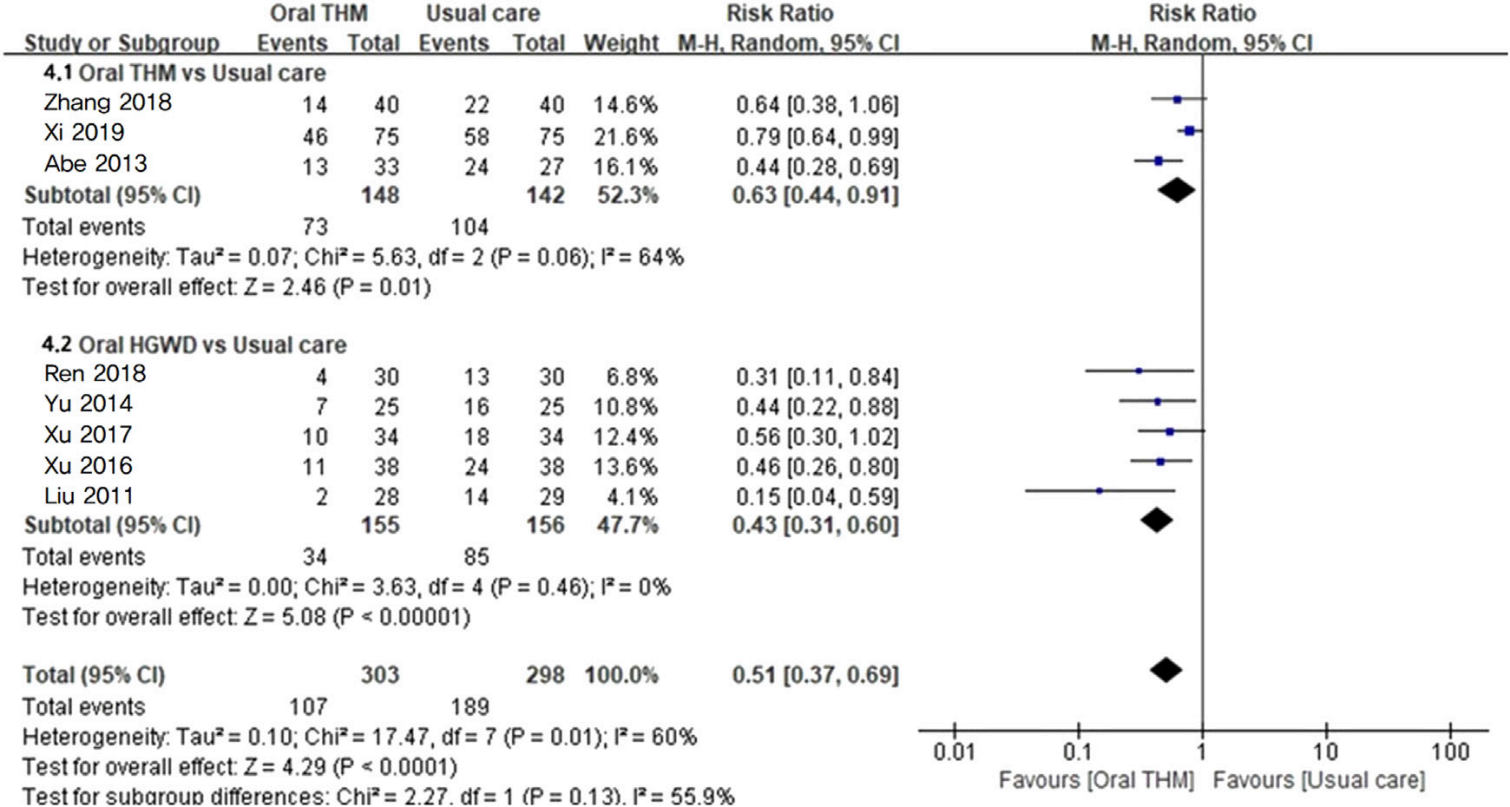
Forest plot of the incidence rate of CIPN: THM *versus* usual care. THM, traditional herbal medicine; HGWD, Huangqi-Guizahi-Wuwu Decoction; CIPN, chemotherapy-induced peripheral neuropathy.

There are currently no pharmacological or non-pharmacological treatments formally recommended as standard interventions for the prevention of CIPN. Therefore, all interventions other than placebo and no treatment—such as vitamin B12 and compression therapy—were classified as usual care. Eight RCTs with 601 participants that reported the incidence rate of CIPN in comparison to usual care to assess the preventive efficacy were included in the meta-analysis (Figure 4) (Liu et al., 2011; Abe et al., 2013; Yu et al., 2014; Xu, 2016; Xu et al., 2017; Ren and Wang, 2018; Zhang Y., 2018; Xi et al., 2019). Overall, THM showed a statistically significant low incidence rate of CIPN compared to usual care (RR 0.51, 95% CI 0.37–0.69; *p* < 0.05), with moderate grade of heterogeneity (*I* ^2^ = 60%). Subgroup analysis revealed that HGWD (RR 0.43, 95% CI 0.31–0.60; *I* ^2^ = 0%; *p* < 0.05) specifically and significantly reduced the incidence rate of CIPN.

The GRADE profile revealed that the quality of evidence for the incidence rate of CIPN with THM compared to usual care was rated as moderate (Table 2).

#### 3.4.3 Incidence rate of CIPN: comparison with no treatment

**FIGURE 5:**
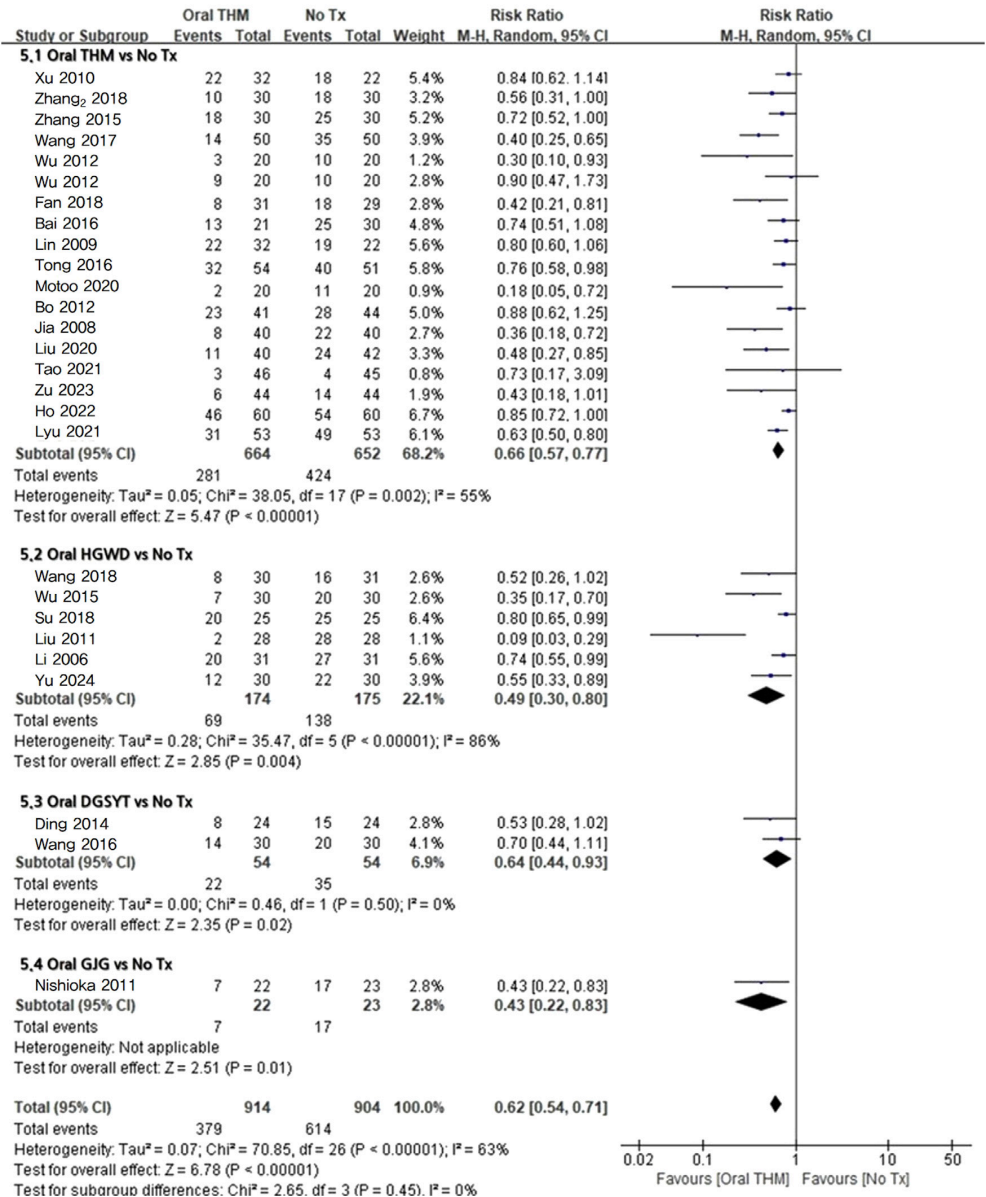
Forest plot of the incidence rate of CIPN: THM *versus* no treatment. THM, traditional herbal medicine; HGWD, Huangqi-Guizahi-Wuwu Decoction; DGSNT, Dang-Gui-Si-Ni-Tang; GJG, Gosha-jinki-gan; CIPN, chemotherapy-induced peripheral neuropathy.

Twenty-six RCTs with 601 participants that reported the incidence rate of CIPN compared no treatment to assess preventive efficacy were included in the meta-analysis, as shown in Figure 5 (Li et al., 2006; Jia et al., 2008; Lin et al., 2009; Xu and Ding, 2010; Liu et al., 2011; Nishioka et al., 2011; Bo and Wenling, 2012; Tao et al., 2012; Wu et al., 2012; Ding et al., 2014; Wu et al., 2015; Zhang et al., 2015; Bai and Shi, 2016; Tong, 2016; Wang Q. et al., 2016; Wang, 2017; Chen et al., 2018; Fan et al., 2018; Su and Huang, 2018; Zhang Y., 2018; Liu et al., 2020; Motoo et al., 2020; Lyu et al., 2021; Ho et al., 2022; Zu et al., 2023; Yu et al., 2024). Overall, THM significantly reduced the incidence rate of CIPN compared to no treatment (RR 0.62, 95% CI 0.54–0.71; *p* < 0.05) with moderate heterogeneity (*I* ^2^ = 63%).

**FIGURE 6:**
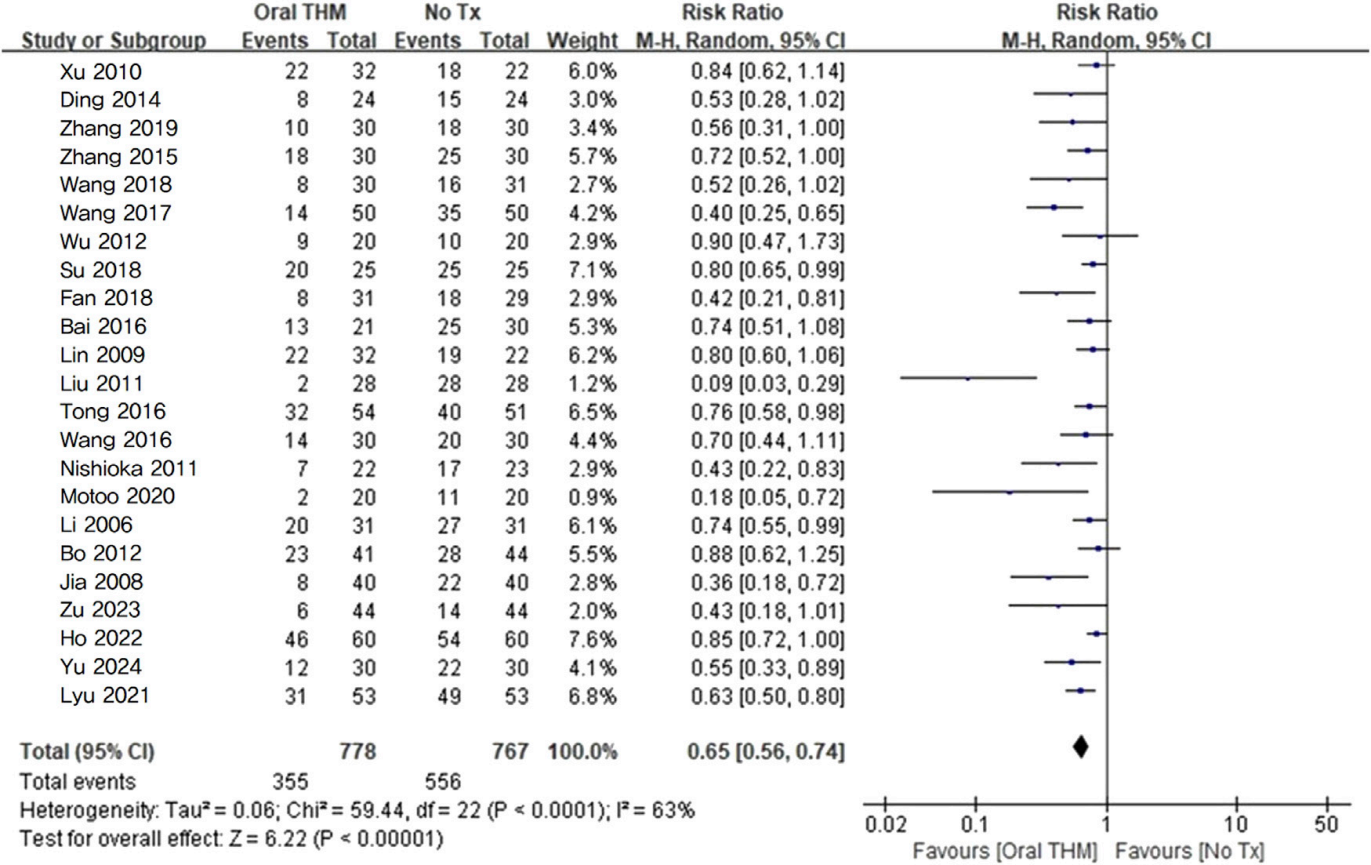
Forest plot of the incidence rate of oxaliplatin-induced CIPN: THM *versus* no treatment. THM, traditional herbal medicine; CIPN, chemotherapy-induced peripheral neuropathy.

Subgroup analysis revealed that HGWD (RR 0.49, 95% CI 0.30–0.80; *p* < 0.05; *I* ^2^ = 86%) and DGSYT (RR 0.64, 95% CI 0.44–0.93; *p* < 0.05; *I* ^2^ = 0%) significantly reduced the incidence rate of CIPN. In addition, the results of a sub-analysis including only studies using oxaliplatin-based CTX (Li et al., 2006; Jia et al., 2008; Lin et al., 2009; Xu and Ding, 2010; Liu et al., 2011; Nishioka et al., 2011; Bo and Wenling, 2012; Wu et al., 2012; Ding et al., 2014; Zhang et al., 2015; Bai and Shi, 2016; Tong, 2016; Wang Q. et al., 2016; Wang, 2017; Chen et al., 2018; Fan et al., 2018; Su and Huang, 2018; Zhang Y., 2018; Motoo et al., 2020; Lyu et al., 2021; Ho et al., 2022; Zu et al., 2023; Yu et al., 2024) revealed that compared with no treatment, THM significantly reduced the incidence rate of oxaliplatin-induced CIPN (RR 0.65, 95% CI 0.56–0.74; *p* < 0.05) with moderate heterogeneity (*I* ^2^ = 63%). In the sub-analysis that assessed the incidence of CIPN using the CTCAE criteria, the pooled risk ratio was 0.57 (95% CI 0.46–0.72; *I* ^2^ = 65%; *p* < 0.05). In the studies that used Levi’s scale for evaluation, the pooled risk ratio was 0.63 (95% CI 0.51–0.77; *I* ^2^ = 67%; *p* < 0.05) (Figure 6).

The GRADE profile revealed that the quality of evidence for the incidence rate of CIPN with THM compared to no treatment was rated as moderate (Table 2).

#### 3.4.4 Incidence rate of CIPN: comparison with all control groups

The overall effect of THM, including all control groups (placebo, usual care, and no treatment), on the incidence rate of CIPN indicated that THM significantly reduced the incidence rate of CIPN compared to all control groups (RR 0.59, 95% CI 0.51–0.68; *p* < 0.05), with high grade of heterogeneity (*I* ^2^ = 84%) (not shown in figure). In the sensitivity analysis excluding studies with a high risk of bias, only the four studies (Kono et al., 2013; Liu et al., 2013; Oki et al., 2015; Cheng et al., 2017) with placebo as the control group remained eligible for meta-analysis, and the results were consistent with those shown in Figure 3 (RR 0.83, 95% CI 0.74–0.93; *I* ^2^ = 91%; *p* < 0.05).

#### 3.4.5 Neuropathic pain intensity

**FIGURE 7:**
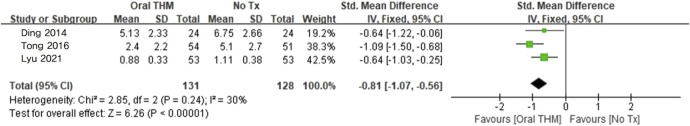
Forest plot of the intensity of neuropathic pain: THM *versus* no treatment. THM, traditional herbal medicine; Tx, treatment.

Three RCTs comparing the efficacy of THM with no treatment involving 259 participants reported the intensity of neuropathic pain using various symptom questionnaires (Ding et al., 2014; Tong, 2016; Lyu et al., 2021). THM showed statistically significant lower pain intensity compared to no treatment (SMD −0.81, 95% CI −1.07 to −0.56; *p* < 0.05), with low grade of heterogeneity (*I* ^2^ = 30%) (Figure 7).

The GRADE profile revealed that the quality of evidence for the intensity of neuropathic pain with THM compared to no treatment was rated as high (Table 2).

#### 3.4.6 KPS

**FIGURE 8:**
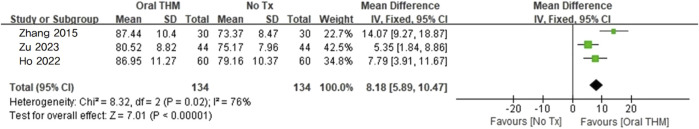
Forest plot of the Karnofsky performance scale: THM *versus* no treatment. THM, traditional herbal medicine; Tx, treatment.

Three RCTs comparing THM with no treatment, involving 268 participants, reported KPS scores to evaluate QoL improvement and were included in the meta-analysis (Figure 8) (Zhang et al., 2015; Ho et al., 2022; Zu et al., 2023). THM significantly improved KPS scores compared to no treatment (MD 8.18, 95% CI 5.89–10.47; *p* < 0.05) with high grade of heterogeneity (*I* ^2^ = 76%).

The GRADE profile indicated that the quality of evidence for KPS improvement with THM compared to no treatment was rated as low due to methodological limitations and inconsistency (Table 2).

#### 3.4.7 NCS parameter

**FIGURE 9:**
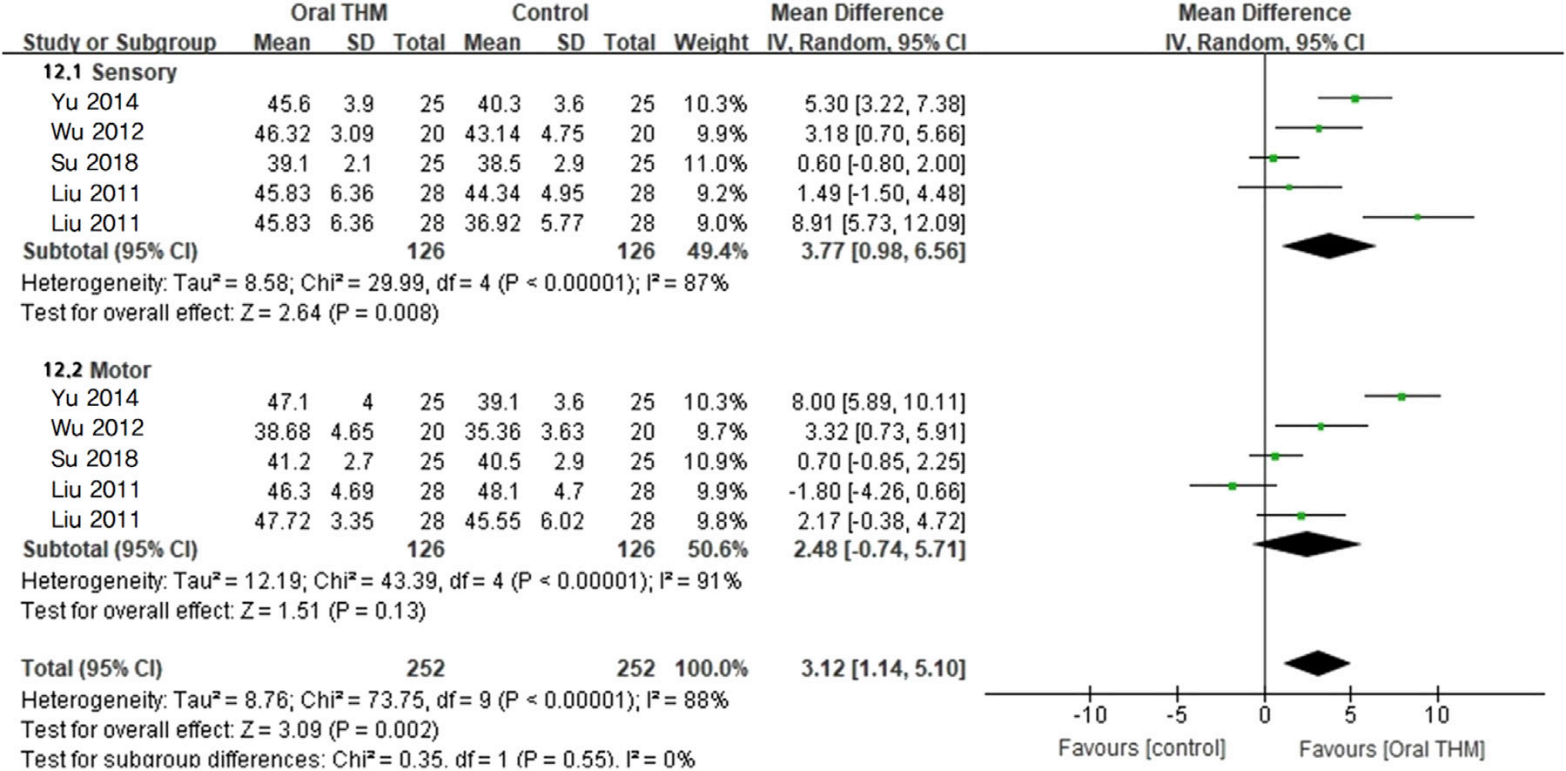
Forest plot of the overall effect of THM on the nerve conduction study (sensory and motor). THM, traditional herbal medicine.

Two RCTs involving 212 participants that compared THM with usual care reported the NCS parameters for the sensory and motor nerves of the peroneal nerve (Liu et al., 2011; Yu et al., 2014). Compared with usual care, treatment with THM resulted in a statistically significant improvement in sensory and motor nerve function (MD 3.94, 95% CI 2.78–5.11; *p* < 0.05), although there was a high grade of heterogeneity (*I* ^2^ = 92%) (not shown in figure). Three RCTs involving 292 participants that compared treatment with THM with no treatment reported the NCS parameters for the sensory and motor nerves of the peroneal nerve (Liu et al., 2011; Wu et al., 2012; Su and Huang, 2018). Compared with no treatment, treatment with THM resulted in a statistically significant improvement in sensory and motor nerve function (MD 1.90, 95% CI 1.08–2.72; *p* < 0.05); however, a high grade of heterogeneity was observed (*I* ^2^ = 81%) (not shown in figure). Figure 9 presents the overall effect of THM on the NCS parameters.

The GRADE profile revealed that the quality of evidence for NCS parameters with THM was rated as low (Table 2).

#### 3.4.8 AEs

Detailed numerical data on the incidence of AEs were lacking in most of the included studies, making it impossible to statistically compare adverse event rates between the two groups. In general, mild symptoms such as anorexia, fatigue, nausea, vomiting, abdominal pain, diarrhea, and neutropenia, were reported in nine studies (Nishioka et al., 2011; Bo and Wenling, 2012; Abe et al., 2013; Kono et al., 2013; Liu et al., 2013; Oki et al., 2015; Cheng et al., 2017; Wang, 2017; Motoo et al., 2020). However, given that all participants underwent CTX concurrently during each trial, these symptoms could not be attributed solely to THM. Notably, no serious AEs, such as liver function abnormalities, were observed during the concurrent treatment processes. A significantly lower incidence of AEs in the THM group compared with that in the no-treatment group was observed in one study (Wang, 2017).

#### 3.4.9 Publication bias

**FIGURE 10:**
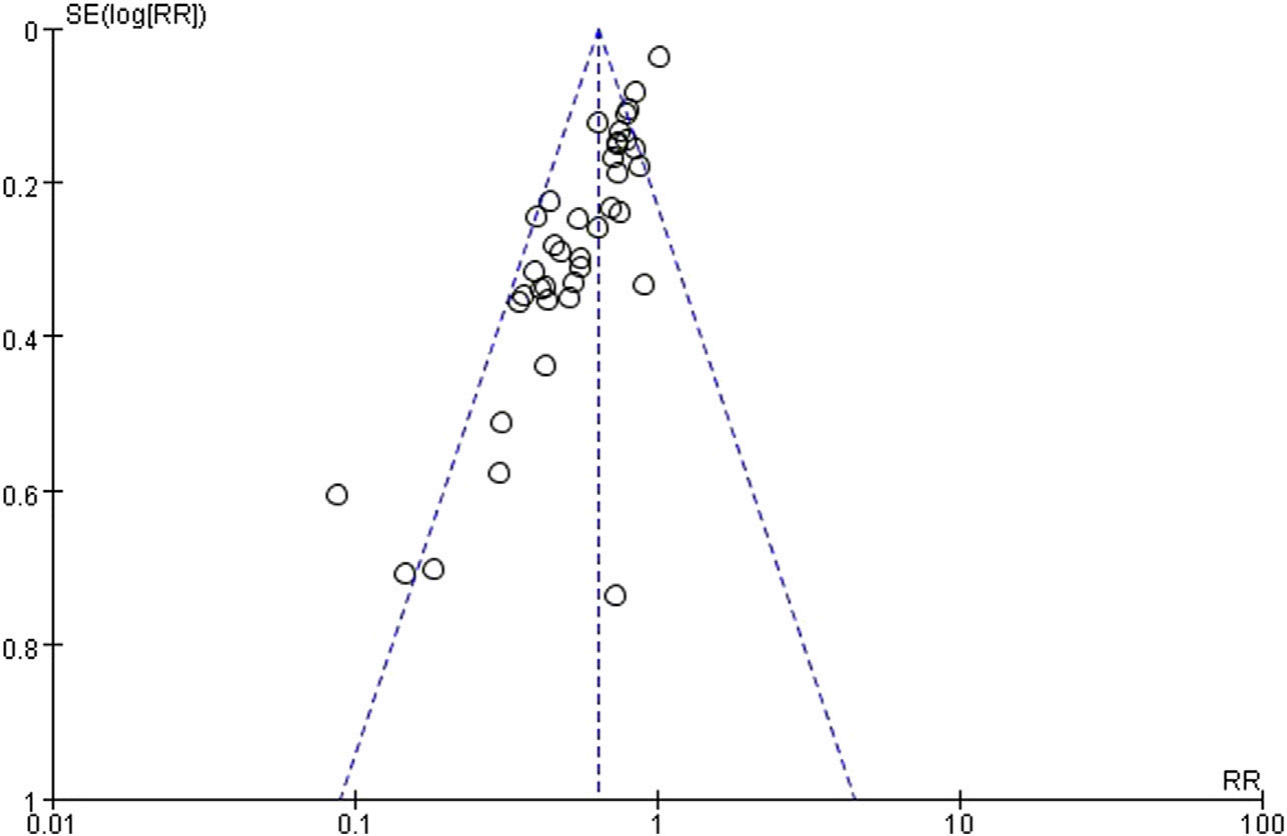
Funnel plot for the meta-analysis of THM for CIPN. THM, traditional herbal medicine; CIPN, chemotherapy-induced peripheral neuropathy.

The funnel plot analysis was conducted to assess the publication bias across all included studies. The plot based on 37 studies exhibited asymmetry, suggesting a potential publication bias. However, further evaluation using Egger’s test yielded a p-value of 0.071 (95% CI, −1.462 to 0.062), indicating no significant evidence of publication bias (Figure 10).

#### 3.4.10 Association rule analysis

A total of 79 herbs were prescribed in the 37 studies included in this review. The cumulative usage frequency of the top ten herbs was 49.57%. The Supplementary Material S2 provides a separate list of herbs used in each study.

The ten most frequently prescribed herbs for the prevention of CIPN were *A. mongholicus Bunge [*Fabaceae*; Astragali Radix]*, *Neolitsea cassia (L.) Kosterm [*Lauraceae*; Cinnamomi Ramulus]*, *Paeonia lactiflora Pall. [*Paeoniaceae*; Paeoniae Radix Alba]*, *Ziziphus jujuba Mill. [*Rhamnaceae*; Zizyphi Fructus]*, *Zingiber officinale Roscoe [*Zingiberaceae*; Zingiberis Rhizoma Recens]*, *Angelica gigas Nakai [*Apiaceae*; Angelicae Gigantis Radix]*, *Glycyrrhiza glabra L. [*Fabaceae*; Glycyrrhizae Radix et Rhizoma]*, *Atractylodes lancea (Thunb.) DC. [*Asteraceae*; Atractylodis Rhizoma Alba]*, *Wolfiporia extensa [*Polyporaceae*; Poria Sclerotium]*, *and Spatholobus suberectus Dunn [*Fabaceae*; Spatholobi Caulis]*. Table 3 presents the frequency distribution of the herbs.

**TABLE 3:** The top 10 herbs prescribed for the prevention of CIPN.

Herb	Frequency of utilization	Relative frequency (%)	Cumulative frequency (%)
*Astragalus mongholicus Bunge [Fabaceae; Astragali Radix]*	27	7.78	7.78
*Neolitsea cassia (L.) Kosterm. [Lauraceae*; *Cinnamomi Ramulus]*	24	6.92	14.70
*Paeonia lactiflora Pall. [Paeoniaceae*; *Paeoniae Radix Alba]*	18	5.19	19.89
*Ziziphus jujuba Mill. [Rhamnaceae*; *Zizyphi Fructus]*	18	5.19	25.08
*Zingiber officinale Roscoe [Zingiberaceae*; *Zingiberis Rhizoma Recens]*	18	5.19	30.27
*Angelica gigas Nakai [Apiaceae*; *Angelicae Gigantis Radix]*	17	4.90	35.17
*Glycyrrhiza glabra L. [Fabaceae*; *Glycyrrhizae Radix et Rhizoma]*	16	4.61	39.78
*Atractylodes lancea (Thunb.) DC. [Asteraceae*; *Atractylodis Rhizoma Alba]*	14	4.03	43.81
*Wolfiporia extensa [Polyporaceae*; *Poria Sclerotium]*	10	2.88	46.69
*Spatholobus suberectus Dunn [Fabaceae*; *Spatholobi Caulis]*	10	2.88	49.57

Abbreviations: CIPN, chemotherapy-induced peripheral neuropathy.

##### 3.4.10.1 A priori algorithm-based association rule analysis

The analysis based on the composition of the 37 included studies (38 THM prescriptions) revealed nine association rules (Supplementary Material S4).

The top herbs with the highest number of association relationships were listed excluding *Z. jujuba Mill. [*Rhamnaceae*; Zizyphi Fructus]*, *Z. officinale Roscoe [*Zingiberaceae*; Zingiberis Rhizoma Recens]*, and *G. glabra L. [*Fabaceae*; Glycyrrhizae Radix et Rhizoma]*, which have shown limited standalone efficacy for the prevention of CIPN in clinical practice: *A. mongholicus Bunge [*Fabaceae*; Astragali Radix]*, *N. cassia (L.) Kosterm [*Lauraceae*; Cinnamomi Ramulus]*, *A. gigas Nakai [*Apiaceae*; Angelicae Gigantis Radix]*, *P. lactiflora Pall. [*Paeoniaceae*; Paeoniae Radix Rubra]*, *A. lancea (Thunb.) DC. [*Asteraceae*; Atractylodis Rhizoma Alba]*, *S. suberectus Dunn [*Fabaceae*; Spatholobi Caulis]*, *W. extensa [*Polyporaceae*; Poria Sclerotium]*, *P. lactiflora Pall [*Paeoniaceae*; Paeoniae Radix Rubra]*, *Rehmannia glutinosa (Gaertn.) Libosch. ex DC [*Orobanchaceae*; Rehmanniae Radix Preparata]*, and *Achyranthes bidentata Blume [*Amaranthaceae*; Achyranthis Radix].*

**FIGURE 11:**
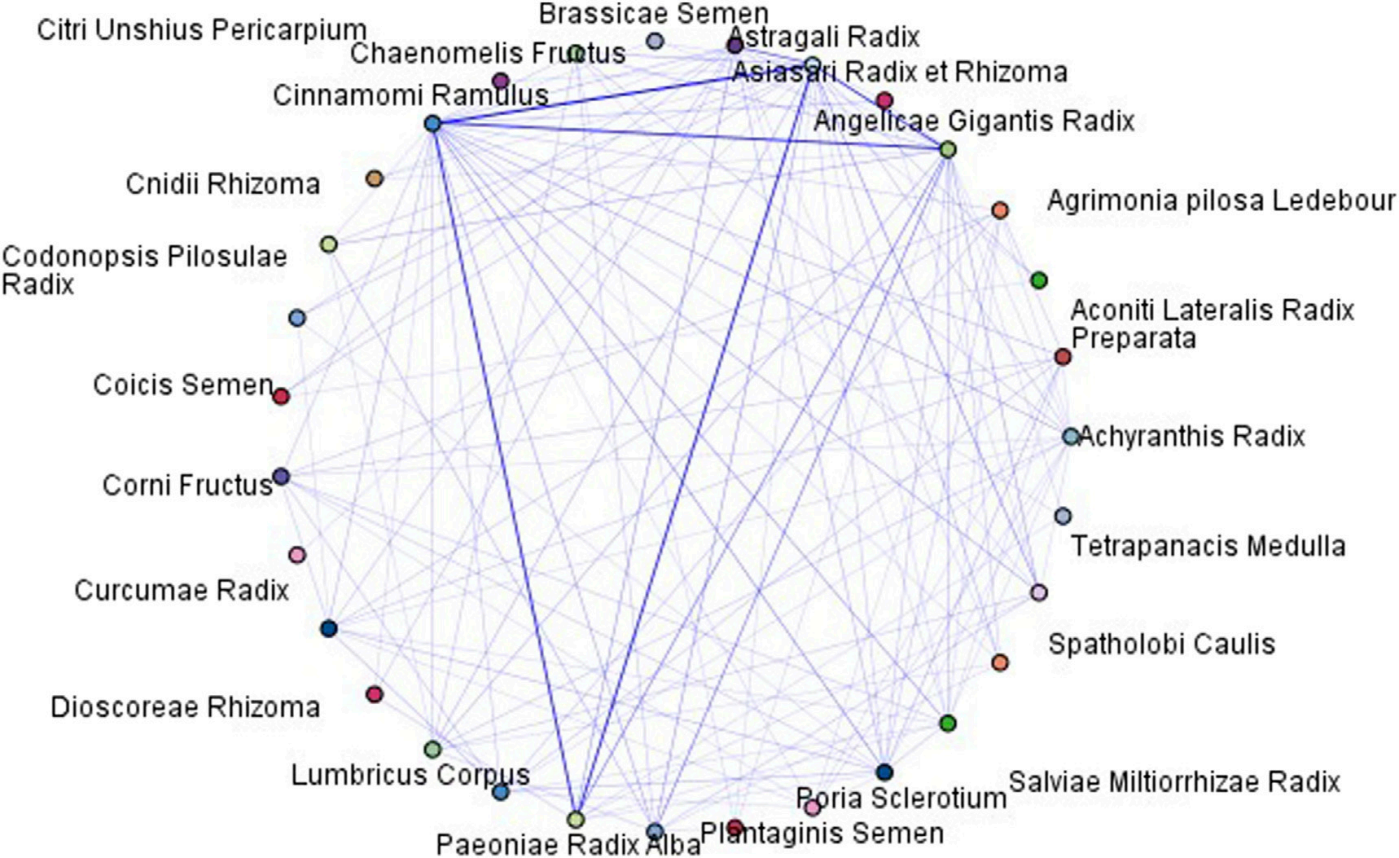
Web chart of herbs included in this study (with a wide threshold).

A web chart, a method within the association rule analysis, was used to visualize the relationships between different herbs. This chart illustrated the likelihood of co-usage of the two herbs in a crossover format. The thicker lines represent stronger correlations. Figure 11 presents a web chart depicting the relationships between the herbs included in the present study.

**FIGURE 12:**
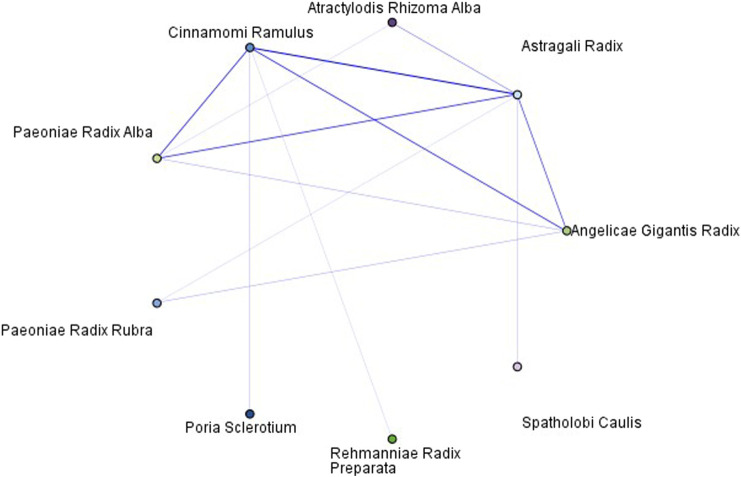
Web chart of herbs included in this study (with a high-reliability threshold).

More distinct correlations can be identified by narrowing the threshold for the reliability of the web chart. The strongest association was observed between *A. mongholicus Bunge [*Fabaceae*; Astragali Radix]*and *N. cassia (L.) Kosterm [*Lauraceae*; Cinnamomi Ramulus]*, followed by the associations between *A. gigas Nakai [*Apiaceae*; Angelicae Gigantis Radix]*and *A. mongholicus Bunge [*Fabaceae*; Astragali Radix]*, *N. cassia (L.) Kosterm [*Lauraceae*; Cinnamomi Ramulus]*, and *P. lactiflora Pall. [*Paeoniaceae*; Paeoniae Radix Alba]*, and *A. mongholicus Bunge [*Fabaceae*; Astragali Radix]*and *P. lactiflora Pall [*Paeoniaceae*; Paeoniae Radix Alba]* (Figure 12).

## 4 Discussion

CIPN, a complication induced by the neurotoxic side effects of CTX agents such as platinum, taxane, and bortezomib, is frequently observed among patients with cancer undergoing CTX. These agents cause CIPN by altering the tertiary structure of neuronal DNA, which results in the deformation of the nerve fiber, destruction of nerve cells, and inhibition of nerve regeneration. Unmanaged CIPN can lead to irreversible sequelae and delay in the commencement of conventional cancer treatment, making early and active management essential (Richardson et al., 2006; Oh and Kim, 2018). Current treatment guidelines recommend the use of duloxetine for the therapeutic treatment of CIPN; however, its use remains limited given its classification as a serotonin-norepinephrine reuptake inhibitor (SNRIs) antidepressant, which may cause adverse effects in older patients and requires close monitoring during prolonged use. Neutropenia, nausea, vomiting, and diarrhea, are common side effects of platinum-based chemotherapy agents, such as oxaliplatin and cisplatin. Nevertheless, they are widely recommended as standard therapeutic drugs and actively used in clinical practice for preventive purposes. The management of CIPN remains challenging (Wickham, 2007; Smith et al., 2013; Fukuda et al., 2017; Loprinzi et al., 2020), particularly with regard to preventive treatment options as a widely accepted pharmacological or non-pharmacological treatment strategy that can be used for preventing CIPN in clinical practice has not been established. Consequently, the analysis of therapeutic treatments for CIPN through systematic reviews and meta-analyses has garnered an increasing amount of attention. However, comprehensive studies that specifically focus on preventive measures are lacking.

This systematic review and meta-analysis with association rule analysis aimed to provide evidence for the preventive efficacy and safety of orally-administered THM in patients with cancer presenting with CIPN. The strength of the present study lies in that the control groups was categorized into three categories—placebo, usual care, and no treatment—and separate meta-analyses were conducted for each of these groups (Jo and Lee, 2021). Furthermore, the present study focused exclusively on orally-administered THM, given their relevance in clinical practice in traditional Korean medicine. It also aimed to identify key herbal combinations that may be associated with preventive effects against CIPN. Thirty-seven studies involving 2,882 patients with cancer scheduled to undergo CTX, which induces peripheral neuropathy as a side effect, were included in the present analysis.

The key findings suggest that THM may have a potential role in reducing the incidence of CIPN compared to various control groups (placebo, usual care, and no treatment). Furthermore, compared with usual care and no treatment, THM was associated with statistically significant improvements in NCS parameters, QoL based on KPS scores, and the intensity of neuropathic pain. Although THM was not associated with a higher incidence of serious adverse events compared to the control interventions, and in some cases showed a more favorable safety profile, it is important to note that most of the included studies—particularly those conducted in China—did not comprehensively report adverse events. THM was administered concurrently with CTX for 4–27 weeks, depending on the planned duration of CTX, which varied according to the type of cancer, stage, and CTX regimen. The absence of serious AEs, including liver function abnormalities, was a noteworthy finding. The association rule analysis revealed that the strongest herbal combination in the included studies was the combination of *A. mongholicus Bunge [*Fabaceae*; Astragali Radix]*and *N. cassia (L.) Kosterm. [*Lauraceae*; Cinnamomi Ramulus]*.

THM had statistically significant therapeutic effects for CIPN compared to usual care or placebo, both when administrated orally (RR 1.67, 95% CI 1.25–2.23, *p* < 0.05; *I* ^2^ = 31%) and topically (RR 2.20, 95% CI 1.52–3.18, *p* < 0.05; *I* ^2^ = 0%) in terms of total effective rate (Kim et al., 2020). The study was particularly significant in that it clarified the potential benefits of THM, distinguishing its routes of administration (oral, topical, washing, or fumigation application), which had previously been conflated in earlier research. However, despite this advancement, subsequent studies involving THM still lack a comprehensive analysis of its preventive effects on CIPN. Given the absence of a standard recommended treatment for the prevention of CIPN, the findings of the present study suggest that orally administered THM may offer potential benefits in reducing the incidence of CIPN compared to clinically used strategies and placebo. Notably, no significant adverse events associated with THM were reported in the included studies. These results, however, should be interpreted with caution due to the high risk of bias and heterogeneity among the included trials. In particular, although the KPS scores improved in the THM group, this outcome is not specific to neuropathy and showed substantial heterogeneity (*I* ^2^ > 80%), which limits the interpretability of the pooled result.

HGWD and GJG are the most commonly used decoctions for the treatment of CIPN in clinical practice. The subgroup analysis conducted herein revealed that compared with usual care and no treatment, THM exhibited significant preventive effects against CIPN when administered orally. A meta-analysis conducted in 2024 (Yang et al., 2024), which focused solely on the ability of HGWD to prevent CIPN, reported a significant reduction in the total incidence of CIPN compared with observed following no treatment (RR 0.57, 95% CI 0.47–0.69; *p* < 0.05; *I* ^2^ = 75%) and usual care (RR 0.58, 95% CI 0.43–0.79; *p* < 0.05; *I* ^2^ = 75%). These findings are generally in line with the results of the present study, suggesting a promising effect of HGWD in reducing the incidence of CIPN. However, a notable distinction in the present study was the method of administration. The administration route of HGWD (oral, fumigation, or external use) was not clearly differentiated when analyzing the preventive efficacy and total effective rate in the meta-analysis conducted by Yang et al. (2024). In contrast, the present study provided a more precise evaluation of the preventive efficacy of HGWD against CIPN by focusing solely on oral administration and excluding concurrent interventions.

In contrast to HGWD, GJG yielded controversial results in previous studies (Yang et al., 2024). A few previous studies assessing its preventive efficacy against CIPN reported that GJG exerted therapeutic effects in patients with grade 3 CIPN (RR 0.42.95% CI 0.25–0.71; *I* ^2^ = 0.0%; *p* < 0.05); however, it exerted no significant effect in patients with grade 2 or higher (RR 0.78, 95% CI 0.36–1.72; *I* ^2^ = 94.7%; *p* = 0.93). The results tended to vary depending on the tools used to evaluate the severity of CIPN, with different outcomes reported for the same severity levels. This inconsistency suggests that the preventive effects of GJG against CIPN are unreliable. Notably, despite control interventions ranging from the administration of a placebo and vitamin B12 to no treatment in a previous study, these were combined into a single group under the control label, and a meta-analysis was conducted based on a single control (Kuriyama and Endo, 2018). In the present study, a subgroup meta-analysis of GJG could not be performed due to the limited number of eligible studies. Nevertheless, the findings suggest that GJG may offer preventive potential against CIPN. Further well-designed studies are warranted to clarify its clinical utility.

A previous meta-analysis demonstrated that THM improved the NCS parameters, reduced the incidence rate, and alleviated the intensity of pain in patients with peripheral neuropathy (PN), including those with CTX-induced, diabetic-induced, and postherpetic neuralgia (Jo and Lee, 2021). Consistent association rules identified *A. mongholicus Bunge [*Fabaceae*; Astragali Radix]*, *N. cassia (L.) Kosterm [*Lauraceae*; Cinnamomi Ramulus]*, and *S. suberectus Dunn [*Fabaceae*; Spatholobi Caulis]* as key constituents of effective herbal combinations. A systematic review published in 2016 identified *Astragali Radix* as a central component of THM decoctions used for the prevention of CIPN, given its significant impact on NCS (Kuriyama and Endo, 2018). In addition to exhibiting a neuroprotective effect by reducing oxidative damage, *N. cassia (L.) Kosterm [*Lauraceae*; Cinnamomi Ramulus]* significantly suppresses the pain hypersensitivity associated with inflammation (Deng et al., 2016; Sun et al., 2016). A combination of *A. mongholicus Bunge [*Fabaceae*; Astragali Radix]*, and *A. gigas Nakai [*Apiaceae*; Angelicae Gigantis Radix]* improves axonal growth by primarily stimulating the neurotrophic signaling pathway in response to central nervous system damage (Zheng et al., 2015). Another study revealed that a combination of *Kosterm [*Lauraceae*; Cinnamomi Ramulus]*, and *G. glabra L. [*Fabaceae*; Glycyrrhizae Radix et Rhizoma]* demonstrated significant differences in the pharmacokinetic parameters compared with the individual use of each herb (Xie et al., 2018). Curcumin, the primary active compound in *Curcuma longa L [*Zingiberaceae*; Curcumae Radix]*, has also been shown to possess neuroprotective properties by reducing oxidative stress and modulating inflammatory responses, as well as protecting against amyloid-beta-induced damage (Sureda et al., 2023). While such pharmacological mechanisms have been elucidated, few studies to date have investigated the mechanistic pathways of the core THM components frequently used in clinical CIPN prevention. Further research is warranted to clarify the neuroprotective effects of these high-frequency herbs and their active constituents.

Thus, rather than focusing solely on the primary mechanisms of individual herbs, the synergistic herbal combinations must be considered to facilitate the effective clinical utilization of THM. The pharmacological activity of THM arises from the synergistic action of multiple chemical components targeting various sites and the simultaneous action of different chemical components targeting a single site (Li et al., 2016; Wang S. et al., 2016; Li and Weng, 2017). The findings of the present study indicate that the combination of *A. mongholicus Bunge [*Fabaceae*; Astragali Radix] and N. cassia (L.) Kosterm. [*Lauraceae*; Cinnamomi Ramulus]* is the strongest herbal combination for preventing the incidence of CIPN, providing valuable evidence for clinical practice in the formulation of THM decoctions.

This review represents the first attempt to evaluate the preventive effects of orally administered THM against CIPN in patients with cancer. The strengths of the present study include the use of rigorous methodologies, such as the PRISMA guidelines and the Cochrane Handbook, along with the assessment of evidence quality using the GRADE profile. Furthermore, the study protocol was registered with PROSPERO, and a comprehensive search was conducted across multiple databases without restrictions on language or country of origin. Although the pooled results suggest that THM may reduce the incidence of CIPN compared to placebo, usual care, and no treatment, the overall effect may be influenced by considerable between-study heterogeneity. Differences in cancer types, CTX regimens, outcome assessment time points, and definitions of CIPN could have contributed to the variability in treatment effects, which may limit the generalizability and precision of the pooled estimates. In addition, substantial heterogeneity was observed across the included studies, including variations in sample sizes, treatment durations, and lack of standardization in the composition and preparation of herbal interventions. Moreover, many studies lacked adequate blinding and reported unclear methods of randomization, which may have led to overestimation of treatment effects. These methodological and clinical differences likely contributed to underlying heterogeneity. As a result, these limitations were reflected in the GRADE assessments, with the quality of evidence for the primary outcome rated as low to moderate. Therefore, although the findings suggest potential preventive benefits of THM, the results should be interpreted with caution due to the high risk of bias and substantial heterogeneity across studies. Despite these limitations, this study is significant in that it specifically focused on the method of administering THM and the categorization of control groups, with separate analyses conducted for each group. These findings may indicate a potential benefit of THM for the prevention of CIPN, particularly in the absence of standard treatment options; however, this interpretation should be made with caution due to the overall low certainty of evidence. Further well-designed, rigorously reported RCTs using standardized methodologies are needed to better establish the clinical utility of THM in this context.

## 5 Conclusion

THM exhibited potential to prevent the incidence of CIPN in patients with cancer. The combination of *A. mongholicus Bunge [*Fabaceae*; Astragali Radix] and N. cassia (L.) Kosterm. [*Lauraceae*; Cinnamomi Ramulus]*, which are among the most commonly used single herbs, was identified as the strongest herbal combination for the prevention of CIPN. However, large-scale, double-blind, randomized controlled trials with rigorous methodological designs must be conducted in the future to definitively determine the efficacy and safety of THM treatment as a strategy for the prevention of CIPN. Future studies must aim to include more generalized populations, standardized herbal ingredients, and appropriate follow-up durations.

## Data Availability

The original contributions presented in the study are included in the article/Supplementary Material, further inquiries can be directed to the corresponding author.

## Abbreviations

AEs, adverse events; CI, confidence intervals; CIPN, chemotherapy-induced peripheral neuropathy; CRC, colorectal cancer; CTX, chemotherapy; DGSNT, Dang-Gui-Si-Ni-Tang; GJG, Gosha-jinki-gan; HGWD, Huangqi-Guizahi-Wuwu Decoction; KPS, Karnofsky performance scale; MD, mean difference; NCS, nerve conduction study; PRISMA, Preferred Reporting Items for Systematic Reviews and Meta-Analysis; PROSPERO, International Prospective Register of Systematic Review; RCT, randomized controlled trial; RR, risk ratios; THM, traditional herbal medicine; QoL, quality of life.
