# Supplementary material for: Traditional herbal medicine for the prevention of chemotherapy-induced peripheral neuropathy: a systematic review and meta-analysis with association rule analysis
Source: Front Pharmacol. 2025 Jun 30;16:1607181. doi: 10.3389/fphar.2025.1607181 (PMC12285655; doi:10.3389/fphar.2025.1607181)
Supplement: Supplementary file 1 [file Supplementaryfile1.docx]

Supplementary Material

#

# S1. Search terms for each database

1. PubMed

#20 #6 and #10 and #19

#19 #11 or #12 or #13 or #14 or #15 or #16 or #17 or #18

#18 (huan qi gui zhi wu wu) or (guilong tongluo) or (qi fu long kui tang) or (wei tiao san hao fang) or (xiao liu tang)

#17 "saikokaryukotsuboreito" [supplementary concept]

#16 "si-jun-zi-tang" [supplementary concept]

#15 "shakuyaku-kanzoh-toh" [supplementary concept]

#14 "buyang huanwu" [supplementary concept]

#13 "gosha-jinki-gan" [supplementary concept]

#12 ("traditional Korean medicine"[tiab] or "traditional Chinese medicine"[tiab] or "Chinese medicine"[tiab] or "traditional oriental medicine"[tiab] or "Kampo medicine"[tiab] or "herb*"[tiab] or "decoction*"[tiab] or "botanic*"[tiab] or "plant*"[tiab] or "extract*"[tiab])

#11 ("Drugs, Chinese Herbal"[MeSH] or "Plants, Medicinal"[MeSH] or "Herbal Medicine"[MeSH] or "Medicine, Kampo"[MeSH] or "Medicine, Korean Traditional"[MeSH] or "Medicine, Chinese Traditional"[MeSH])

#10 #7 or #8 or #9

#9 (neuropath* or neuralgi* or peripheral neuropathy or sensory impairment or neurotoxicity or CIPN)

#8 "Neuralgia"[Mesh]

#7 "Peripheral nervous system diseases"[Mesh]

#6 #1 or #2 or #3 or #4 or #5

#5 (chemotherapeutic drug or chemotherapy or cisplatin* or oxaliplatin or taxane or thalidomide or bortezomib*)

#4 "Antineoplastic agents"[Mesh]

#3 "Drug therapy"[Mesh]

#2 (neoplasm* or cancer* or carcino* or malignan* or tumor* or tumour*)

#1 “Neoplasms"[Mesh]

2. EMBASE

#14 #4 and #8 and #12 and #13

#13 [cochrane review]/lim or [systematic review]/lim or [meta analysis]/lim or [controlled clinical trial]/lim or [randomized controlled trial]/lim

#12 #9 or #10 or #11

#11 'goshajinkigan' or 'rikkunshito' or 'saiko ka ryukotsu borei to' or 'huan qi gui zhi wu' or 'guilong tongluo' or 'buyang huanwu' or 'shao yao gan' or 'qi fu long kui tang' or 'wei tiao san hao fang' or 'xiao liu tang'

#10 herb* or extract* or plant*

#9 'chinese medicine' or 'korean medicine' or 'kampo medicine'

#8 #5 or #6 or #7

#7 (peripheral and ('neuropathy'/exp or neuropathy)) or (('sensory'/exp or sensory) and ('impairment'/exp or impairment)) or CIPN or neurotoxicity)

#6 'neuralgia'/exp or 'neuralgia' or neuropath* or neuralgi*

#5 'peripheral neuropathy'/exp or 'peripheral neuropathy'

#4 #1 or #2 or #3

#3 cisplatin* or 'oxaliplatin'/exp or 'taxane'/exp or 'thalidomide'/exp or bortezomib*

#2 'drug therapy'/exp or 'antineoplastic agent'/exp or 'chemotherapy'/exp or (chemotherapeutic AND ('drug'/exp OR drug))

#1 'neoplasm' or 'neoplasm'/exp or neoplasms or neoplasm* or cancer* or carcino* or malignan* or tumor* or tumour*

3. Cochrane Library

#1 MeSH descriptor: [Neoplasms] explode all trees

#2 neoplasm* or cancer* or carcino* or malignan* or tumor* or tumour*

#3 MeSH descriptor: [Drug Therapy] explode all trees

#4 chemotherapy or chemotherapeutic drug or cisplatin* or oxaliplatin or taxane or thalidomide or bortezomib*

#5 #1 or #2 or #3 or #4

#6 MeSH descriptor: [Peripheral Nervous System Diseases] explode all trees

#7 neuropath* or neuralgi* or peripheral neuropathy or sensory impairment or neurotoxicity or CIPN

#8 #6 or #7

#9 MeSH descriptor: [Medicine, Chinese Traditional] explode all trees

#10 MeSH descriptor: [Medicine, Korean Traditional] explode all trees

#11 MeSH descriptor: [Medicine, Kampo] explode all trees

#12 Chinese medicine or herb* or extract* or plant* or decoction*

#13 'goshajinkigan' or 'buyang huanwu' or 'shao yao gan cao tang' or 'sijunzi' or 'saikokaryukotsuboreito' or 'huan qi gui zhi wu wu' or 'guilong tongluo' or 'qi fu long kui tang' or 'wei tiao san hao fang' or 'xiao liu tang'

#14 #9 or #10 or #11 or #12 or #13

#15 #5 and #8 and #14

#16 #15 in trials

4. CNKi

#1 癌

#2 肿瘤

#3 cancer

#4 tumor

#5 neoplasm

#6 药物治疗

#7 化疗

#8 chemotherapy

#9 chemotherapeutic drug

#10 cisplatin

#11 oxaliplatin

#12 taxane

#13 thalidomide

#14 bortezomib

#15 xeloda

#16 OR / 1-15

#17 病变

#18 神经痛

#19 周围神经病变

#20 neuropathy

#21 neuralgia

#22 neurotoxicity

#23 CIPN

#24 sensory impairment

#25 OR / 17-24

#26 中药

#27 中医

#28 汤

#29 饮

#30 散

#31 汤剂

#32 丸

#33 中成药

#34 方剂

#35 中西医结合

#36 颗粒

#37 胶囊

#38 口服液

#39 Capsule

#40 Powder

#41 Herbal medicine

#42 Traditional Chinese medicine

#43 TCM

#44 Traditional medicine

#45 Decoction

#46 Chinese medicine

#47 牛车肾气丸

#48 补阳还五汤

#49 芍药甘草汤

#50 四君子汤

#51 柴胡龙骨牡蛎汤

#52 黄芪桂枝五物汤

#53 桂龙通络

#54 芪附龙葵汤

#55 微调3号方

#56 消瘤汤

#57 goshajinkigan

#58 saikokaryukotsuboreito

#59 sijunzi

#60 buyang huanwu

#61 qi fu long kui tang

#62 wei tiao san hao fang

#63 xiao liu tang

#64 OR / 26-63

#65 随机

#66 对照

#67 Randomized trials

#68 系统评价

#69 Systematic reviews

#70 Meta分析

#71 clinical

#72 trial

#73 research

#74 random

#75 randomized

#76 OR / 65-75

#77 #16 AND #25 AND #64 AND #76

5. CiNii

#1 (cancer OR neoplasms OR drug therapy OR chemotherapy OR 癌 OR 新生物 OR 薬治療 OR 化学療法) AND (neuropathy OR neuralgia OR 神経障害 OR 神経痛) AND (Kampo OR herb OR extract OR plant OR decoction OR traditional medicine)

#2 randomized controlled trial OR trial OR 対照比較試験 OR 試み

#3 #1 AND #2

6. KMBASE

#1 ([KEYWORD=cancer] OR [KEYWORD=neoplasms] OR [KEYWORD=drug therapy] OR [KEYWORD=chemotherapy])

#2 ([ABSTRACT=neuropathy] OR [ABSTRACT=neuralgia] OR [ALL=CIPN])

#3 (([ABSTRACT=traditional medicine] OR [ABSTRACT=korean medicine]) OR [ABSTRACT=herb])

#4 ((((([KEYWORD=cancer] OR [ALL=neoplasms]) OR [ALL=drug therapy]) OR [ALL=chemotherapy]) AND (([ABSTRACT=neuropathy] OR [ABSTRACT=neuralgia]) OR [ALL=CIPN])) AND ((([ABSTRACT=traditional medicine] OR [ABSTRACT=korean medicine]) OR [ABSTRACT=herb]) OR [ABSTRACT=decoction]))

7. KISS

#1 cancer AND neuropathy AND Korean medicine

#2 cancer AND neuropathy AND traditional medicine

#3 cancer AND neuropathy AND herb

#4 OR / 1-3

8. NDSL

#1 ABSTRACT=cancer | neoplasm

#2 ABSTRACT =neuropathy | neuralgia | CIPN

#3 ABSTRACT =Korean medicine | traditional medicine | herb

#4 ABSTRACT =randomized

#5 AND / 1-4

9. OASIS

#1 cancer AND neuropathy AND Korean medicine

#2 cancer AND neuropathy AND traditional medicine

#3 cancer AND neuropathy AND herb

#4 OR / 1-3

# S2. The GRADE assessment for each PICO question

Risk of Bias, if no serious bias were observed across the included studies, it was marked as “not serious.” When serious bias was observed in one domain (e.g., blinding or allocation concealment), it was rated as “serious,” and if two or more domains showed major bias, it was classified as “very serious.”

Inconsistency was evaluated based on heterogeneity statistics. An I² value between 0% and 49% was considered “not serious,” 50–75% as “serious,” and greater than 75% as “very serious.”

Indirectness was rated “not serious” when the study populations, interventions, comparators, and outcomes directly answered the research question. Otherwise, it was rated “serious” or “very serious” depending on the degree of indirectness.

Imprecision was judged by examining confidence intervals. If the confidence interval did not cross the null effect (0 for MD/SMD or 1 for RR) and the p-value was <0.05, it was deemed “not serious.” If the interval crossed the null or the sample size was small (total n <100 in meta-analysis), it was considered “serious.”

Other considerations, such as publication bias, were also assessed. In the absence of specific concerns, it was rated as “not serious”; otherwise, “serious” or “very serious” was applied based on the extent of bias.

# S3. Supplementary Table 2

# Table 1. Herbal formulas and its components in the included studies.

| Study  (Year) | Name of prescription | Composition | Ratios of the  Components (%) |
| --- | --- | --- | --- |
| Oki  (2015) | GJG | *Achyranthes bidentata Blume [Amaranthaceae; Achyranthis Radix]*  *Cornus officinalis Siebold & Zucc. [Cornaceae; Corni Fructus]*  *Wolfiporia extensa [Polyporaceae; Poria Sclerotium]*  *Paeonia × suffruticosa Andrews [Paeoniaceae; Moutan Radicis Cortex]*  *Dioscorea oppositifolia L. [Dioscoreaceae; Dioscoreae Rhizoma]*  *Plantago asiatica L. [Plantaginaceae; Plantaginis Semen]*  *Neolitsea cassia (L.) Kosterm. [Lauraceae; Cinnamomi Ramulus]*  *Rehmannia glutinosa (Gaertn.) Libosch. ex DC. [Orobanchaceae; Rehmanniae Radix Preparata]*  *Aconitum carmichaelii Debeaux [Ranunculaceae; Aconiti Lateralis Radix Preparata]*  *Alisma plantago-aquatica subsp. orientale (Sam.) Sam. [Alismataceae; Alismatis Rhizoma]* | 11  11  11  11  11  11  3  17  3  11 |
| Liu  (2013) | Tong-luo fang | *Neolitsea cassia (L.) Kosterm. [Lauraceae; Cinnamomi Ramulus]*  *Astragalus mongholicus Bunge [Fabaceae; Astragali Radix]*  *Pheretima communissima [Lumbricidae; Lumbricus Corpus]*  *Carthamus tinctorius L. [Asteraceae; Carthami Flos]*  *Angelica gigas Nakai [Apiaceae; Angelicae Gigantis Radix]*  *Conioselinum anthriscoides 'Chuanxiong' [Apiaceae; Cnidii Rhizoma]*  *Spatholobus suberectus Dunn [Fabaceae; Spatholobi Caulis]*  *Paeonia lactiflora Pall. [Paeoniaceae; Paeoniae Radix Alba]*  *Curcuma longa L. [Zingiberaceae; Curcumae Radix]*  *Glycyrrhiza glabra L. [Fabaceae;Glycyrrhizae Radix et Rhizoma]* | 5  19  8  5  8  8  19  19  5  4 |
| Kono  (2013) | GJG | *Achyranthes bidentata Blume [Amaranthaceae; Achyranthis Radix]*  *Cornus officinalis Siebold & Zucc. [Cornaceae; Corni Fructus]*  *Wolfiporia extensa [Polyporaceae; Poria Sclerotium]*  *Paeonia × suffruticosa Andrews [Paeoniaceae; Moutan Radicis Cortex]*  *Dioscorea oppositifolia L. [Dioscoreaceae; Dioscoreae Rhizoma]*  *Plantago asiatica L. [Plantaginaceae; Plantaginis Semen]*  *Neolitsea cassia (L.) Kosterm. [Lauraceae; Cinnamomi Ramulus]*  *Rehmannia glutinosa (Gaertn.) Libosch. ex DC. [Orobanchaceae; Rehmanniae Radix Preparata]*  *Aconitum carmichaelii Debeaux [Ranunculaceae; Aconiti Lateralis Radix Preparata]*  *Alisma plantago-aquatica subsp. orientale (Sam.) Sam. [Alismataceae; Alismatis Rhizoma]* | 11  11  11  11  11  11  3  17  3  11 |
| Cheng  (2017) | HGWD | *Astragalus mongholicus Bunge [Fabaceae; Astragali Radix]*  *Neolitsea cassia (L.) Kosterm. [Lauraceae; Cinnamomi Ramulus]*  *Paeonia lactiflora Pall. [Paeoniaceae; Paeoniae Radix Alba]*  *Zingiber officinale Roscoe [Zingiberaceae; Zingiberis Rhizoma Recens]*  *Ziziphus jujuba Mill. [Rhamnaceae;Zizyphi Fructus]* | 32  17  17  17  17 |
| Zhang  (2018) | Decoction for individual research | *Astragalus mongholicus Bunge [Fabaceae; Astragali Radix]*  *Lycium chinense Mill. [Solanaceae; Lycii Fructus]*  *Rehmannia glutinosa (Gaertn.) Libosch. ex DC. [Orobanchaceae; Rehmanniae Radix Recens]*  *Spatholobus suberectus Dunn [Fabaceae; Spatholobi Caulis]*  *Neolitsea cassia (L.) Kosterm. [Lauraceae; Cinnamomi Ramulus]*  *Pheretima communissima [Lumbricidae; Lumbricus Corpus]*  *Carthamus tinctorius L. [Asteraceae; Carthami Flos]*  *Asarum heterotropoides F.Schmidt [Aristolochiaceae; Asiasari Radix et Rhizoma]*  *Pueraria montana var. lobata (Willd.) Maesen & S.M.Almeida ex Sanjappa & Predeep [Fabaceae; Pueraria Root]*  *Achyranthes bidentata Blume [Amaranthaceae; Achyranthis Radix]* | 17  11  9  9  9  5  11  9  11  9 |
| Ren  (2018) | HGWD | *Astragalus mongholicus Bunge [Fabaceae; Astragali Radix]*  *Neolitsea cassia (L.) Kosterm. [Lauraceae; Cinnamomi Ramulus]*  *Paeonia lactiflora Pall. [Paeoniaceae; Paeoniae Radix Rubra]*  *Salvia miltiorrhiza Bunge [Lamiaceae; Salviae Miltiorrhizae Radix]*  *Angelica gigas Nakai [Apiaceae; Angelicae Gigantis Radix]*  *Pheretima communissima [Lumbricidae; Lumbricus Corpus]*  *Conioselinum anthriscoides ‘Chuanxiong’ [Apiaceae; Cnidii Rhizoma]*  *Zingiber officinale Roscoe [Zingiberaceae; Zingiberis Rhizoma Recens]*  *Glycyrrhiza glabra L. [Fabaceae; Glycyrrhizae Radix et Rhizoma]* | 36  8  8  8  8  8  8  8  8 |
| Yu  (2014) | HGWD | *Astragalus mongholicus Bunge [Fabaceae; Astragali Radix]*  *Neolitsea cassia (L.) Kosterm. [Lauraceae; Cinnamomi Ramulus]*  *Paeonia lactiflora Pall. [Paeoniaceae; Paeoniae Radix Alba]*  *Zingiber officinale Roscoe [Zingiberaceae; Zingiberis Rhizoma Recens]*  *Ziziphus jujuba Mill. [Rhamnaceae; Zizyphi Fructus]* | 38  19  19  19  5 |
| Xi  (2019) | Decoction for strengthen the spleen | *Vitis vinifera L. [Vitaceae; Vitis vinifera]*  *Wolfiporia extensa [Polyporaceae; Poria Sclerotium]*  *Astragalus mongholicus Bunge [Fabaceae; Astragali Radix]*  *Wisteriopsis reticulata (Benth.) J.Compton & Schrire [Fabaceae; Millettiae Caulis]*  *Atractylodes lancea (Thunb.) DC. [Asteraceae; Atractylodis Rhizoma Alba] Codonopsis pilosula (Franch.) Nannf. [Campanulaceae; Codonopsis Pilosulae Radix]* | 19  19  19  19  11  13 |
| Xu  (2017) | HGWD | *Astragalus mongholicus Bunge [Fabaceae; Astragali Radix]*  *Spatholobus suberectus Dunn [Fabaceae; Spatholobi Caulis]*  *Codonopsis pilosula (Franch.) Nannf. [Campanulaceae; Codonopsis Pilosulae Radix]*  *Neolitsea cassia (L.) Kosterm. [Lauraceae; Cinnamomi Ramulus]*  *Angelica gigas Nakai [Apiaceae; Angelicae Gigantis Radix]*  *Asarum heterotropoides F.Schmidt [Aristolochiaceae; Asiasari Radix et Rhizoma]*  *Glycyrrhiza glabra L. [Fabaceae; Glycyrrhizae Radix et Rhizoma]*  *Zingiber officinale Roscoe [Zingiberaceae; Zingiberis Rhizoma Recens]*  *Ziziphus jujuba Mill. [Rhamnaceae; Zizyphi Fructus]* | 24  24  12  8  8  2  5  8  9 |
| Xu  (2016) | HGWD | *Paeonia lactiflora Pall. [Paeoniaceae; Paeoniae Radix Alba]*  *Neolitsea cassia (L.) Kosterm. [Lauraceae; Cinnamomi Ramulus]*  *Astragalus mongholicus Bunge [Fabaceae; Astragali Radix]*  *Ziziphus jujuba Mill. [Rhamnaceae; Zizyphi Fructus]*  *Zingiber officinale Roscoe [Zingiberaceae; Zingiberis Rhizoma Recens]* | 17  17  35  8  23 |
| Liu  (2011) | HGWD | *Astragalus mongholicus Bunge [Fabaceae; Astragali Radix]*  *Neolitsea cassia (L.) Kosterm. [Lauraceae; Cinnamomi Ramulus]*  *Paeonia lactiflora Pall. [Paeoniaceae; Paeoniae Radix Alba]*  *Zingiber officinale Roscoe [Zingiberaceae; Zingiberis Rhizoma Recens]*  *Ziziphus jujuba Mill. [Rhamnaceae; Zizyphi Fructus]* | 36  14  18  14  18 |
| Abe  (2013) | GJG | *Achyranthes bidentata Blume [Amaranthaceae; Achyranthis Radix]*  *Cornus officinalis Siebold & Zucc. [Cornaceae; Corni Fructus]*  *Wolfiporia extensa [Polyporaceae; Poria Sclerotium]*  *Paeonia × suffruticosa Andrews [Paeoniaceae; Moutan Radicis Cortex]*  *Dioscorea oppositifolia L. [Dioscoreaceae; Dioscoreae Rhizoma]*  *Plantago asiatica L. [Plantaginaceae; Plantaginis Semen]*  *Neolitsea cassia (L.) Kosterm. [Lauraceae; Cinnamomi Ramulus]*  *Rehmannia glutinosa (Gaertn.) Libosch. ex DC. [Orobanchaceae; Rehmanniae Radix Preparata]*  *Aconitum carmichaelii Debeaux [Ranunculaceae; Aconiti Lateralis Radix Preparata]*  *Alisma plantago-aquatica subsp. orientale (Sam.) Sam. [Alismataceae; Alismatis Rhizoma]* | 11  11  11  11  11  11  3  17  3  11 |
| Xu  (2010) | Yiqi Huoxue Decoction | *Astragalus mongholicus Bunge [Fabaceae; Astragali Radix]*  *Atractylodes lancea (Thunb.) DC. [Asteraceae; Atractylodis Rhizoma Alba]*  *Coix lacryma-jobi var. ma-yuen (Rom.Caill.) Stapf [Poaceae; Coicis Semen]*  *Agrimonia pilosa Ledeb. [Rosaceae; Agrimonia pilosa Ledebour]*  *Salvia miltiorrhiza Bunge [Lamiaceae; Salviae Miltiorrhizae Radix]*  *Spatholobus suberectus Dunn [Fabaceae; Spatholobi Caulis]*  *Reynoutria japonica Houtt. [Polygonaceae; Polygoni Cuspidati Radix]* | No details |
| Ding  (2014) | DGSNT | *Angelica gigas Nakai [Apiaceae; Angelicae Gigantis Radix]*  *Neolitsea cassia (L.) Kosterm. [Lauraceae; Cinnamomi Ramulus]*  *Asarum heterotropoides F.Schmidt [Aristolochiaceae; Asiasari Radix et Rhizoma]*  *Paeonia lactiflora Pall. [Paeoniaceae; Paeoniae Radix Alba]*  *Tetrapanax papyrifer (Hook.) K.Koch [Araliaceae; Tetrapanacis Medulla]*  *Glycyrrhiza glabra L. [Fabaceae; Glycyrrhizae Radix et Rhizoma]*  *Ziziphus jujuba Mill. [Rhamnaceae; Zizyphi Fructus]* | 20  20  6  22  20  6  6 |
| Zhang_2_  (2018) | Yanghe Decoction | *Rehmannia glutinosa (Gaertn.) Libosch. ex DC. [Orobanchaceae; Rehmanniae Radix Preparata]*  *Astragalus mongholicus Bunge [Fabaceae; Astragali Radix]*  *Neolitsea cassia (L.) Kosterm. [Lauraceae; Cinnamomi Ramulus]*  *Angelica gigas Nakai [Apiaceae; Angelicae Gigantis Radix]*  *Cervus elaphus Linnaeus [Cervidae; Cervi Cornu]*  *Ephedra sinica Stapf [Ephedraceae;Ephedrae Herba]*  *Brassica rapa L. [Brassicacea; Brassicae Semen]*  *Tetrapanax papyrifer (Hook.) K.Koch [Araliaceae; Tetrapanacis Medulla]*  *Zingiber officinale Roscoe [Zingiberaceae; Zingiberis Rhizoma Recens]*  *Paeonia lactiflora Pall. [Paeoniaceae; Paeoniae Radix Alba]*  *Glycyrrhiza glabra L. [Fabaceae; Glycyrrhizae Radix et Rhizoma]*  *Ziziphus jujuba Mill. [Rhamnaceae; Zizyphi Fructus]* | 22  22  8  10  7  3  5  2  1  10  2  2 |
| Zhang  (2015) | Bazhen Decoction | *Angelica gigas Nakai [Apiaceae; Angelicae Gigantis Radix]*  *Rehmannia glutinosa (Gaertn.) Libosch. ex DC. [Orobanchaceae; Rehmanniae Radix Preparata]*  *Paeonia lactiflora Pall. [Paeoniaceae; Paeoniae Radix Alba]*  *Conioselinum anthriscoides ‘Chuanxiong’ [Apiaceae; Cnidii Rhizoma]*  *Atractylodes lancea (Thunb.) DC. [Asteraceae; Atractylodis Rhizoma Alba]*  *Panax ginseng C.A.Mey. [Araliaceae; Ginseng Radix]*  *Neolitsea cassia (L.) Kosterm. [Lauraceae; Cinnamomi Ramulus]*  *Glycyrrhiza glabra L. [Fabaceae; Glycyrrhizae Radix et Rhizoma]*  *Wolfiporia extensa [Polyporaceae; Poria Sclerotium]*  *Zingiber officinale Roscoe [Zingiberaceae; Zingiberis Rhizoma Recens]* | 14  14  8  10  8  4  10  8  14  10 |
| Wang  (2018) | HGWD | *Astragalus mongholicus Bunge [Fabaceae; Astragali Radix]*  *Neolitsea cassia (L.) Kosterm. [Lauraceae; Cinnamomi Ramulus]*  *Paeonia lactiflora Pall. [Paeoniaceae; Paeoniae Radix Alba]*  *Zingiber officinale Roscoe [Zingiberaceae; Zingiberis Rhizoma Recens]*  *Ziziphus jujuba Mill. [Rhamnaceae; Zizyphi Fructus]* | 42  6  28  14  10 |
| Wang  (2017) | Lizhong Decoction | *Os draconis nativus [Elephantidae; Fossilia Ossis Mastodi]*  *Ostrea gigas Thunberg [Ostreidae;Ostreae Testa]*  *Lablab purpureus subsp. purpureus [Fabaceae; Dolichoris Semen]*  *Dioscorea oppositifolia L. [Dioscoreaceae; Dioscoreae Rhizoma]*  *Zingiber officinale Roscoe [Zingiberaceae; Zingiberis Rhizoma Recens]*  *Panax ginseng C.A.Mey. [Araliaceae; Ginseng Radix]*  *Atractylodes lancea (Thunb.) DC. [Asteraceae; Atractylodis Rhizoma Alba]*  *Paeonia lactiflora Pall. [Paeoniaceae; Paeoniae Radix Alba]*  *Neolitsea cassia (L.) Kosterm. [Lauraceae; Cinnamomi Ramulus]*  *Ziziphus jujuba Mill. [Rhamnaceae; Zizyphi Fructus]*  *Glycyrrhiza glabra L. [Fabaceae; Glycyrrhizae Radix et Rhizoma]* | 19  19  10  10  7  7  7  7  5  5  4 |
| Wu  (2015) | HGWD | *Astragalus mongholicus Bunge [Fabaceae; Astragali Radix]*  *Paeonia lactiflora Pall. [Paeoniaceae; Paeoniae Radix Rubra]*  *Neolitsea cassia (L.) Kosterm. [Lauraceae; Cinnamomi Ramulus]*  *Zingiber officinale Roscoe [Zingiberaceae; Zingiberis Rhizoma Recens]*  *Ziziphus jujuba Mill. [Rhamnaceae; Zizyphi Fructus]*  *Spatholobus suberectus Dunn [Fabaceae; Spatholobi Caulis]*  *Angelica gigas Nakai [Apiaceae; Angelicae Gigantis Radix]*  *Conioselinum anthriscoides ‘Chuanxiong’ [Apiaceae; Cnidii Rhizoma]* | No details |
| Wu  (2012) | Bu-yang-huan-wu-tang | *Astragalus mongholicus Bunge [Fabaceae; Astragali Radix]*  *Angelica gigas Nakai [Apiaceae; Angelicae Gigantis Radix]*  *Paeonia lactiflora Pall. [Paeoniaceae; Paeoniae Radix Rubra]*  *Conioselinum anthriscoides ‘Chuanxiong’ [Apiaceae; Cnidii Rhizoma]*  *Prunus persica (L.) Batsch [Rosaceae; Persicae Semen]*  *Carthamus tinctorius L. [Asteraceae; Carthami Flos]*  *Pheretima communissima [Lumbricidae; Lumbricus Corpus]* | 52  8  8  8  8  8  8 |
| Wu  (2012) | Sijunzi Decoction plus Shingi-whan | *Codonopsis pilosula (Franch.) Nannf. [Campanulaceae; Codonopsis Pilosulae Radix]*  *Atractylodes lancea (Thunb.) DC. [Asteraceae; Atractylodis Rhizoma Alba]*  *Wolfiporia extensa [Polyporaceae; Poria Sclerotium]*  *Glycyrrhiza glabra L. [Fabaceae; Glycyrrhizae Radix et Rhizoma]*  *Rehmannia glutinosa (Gaertn.) Libosch. ex DC. [Orobanchaceae; Rehmanniae Radix Preparata]*  *Dioscorea oppositifolia L. [Dioscoreaceae; Dioscoreae Rhizoma]*  *Cornus officinalis Siebold & Zucc. [Cornaceae; Corni Fructus]*  *Alisma plantago-aquatica subsp. orientale (Sam.) Sam. [Alismataceae; Alismatis Rhizoma]*  *Paeonia × suffruticosa Andrews [Paeoniaceae; Moutan Radicis Cortex]*  *Neolitsea cassia (L.) Kosterm. [Lauraceae; Cinnamomi Ramulus]*  *Aconitum carmichaelii Debeaux [Ranunculaceae; Aconiti Lateralis Radix Preparata]* | 12  9  9  6  14  12  12  9  9  6  2 |
| Su  (2018) | HGWD | *Astragalus mongholicus Bunge [Fabaceae; Astragali Radix]*  *Neolitsea cassia (L.) Kosterm. [Lauraceae; Cinnamomi Ramulus]*  *Paeonia lactiflora Pall. [Paeoniaceae; Paeoniae Radix Alba]*  *Zingiber officinale Roscoe [Zingiberaceae; Zingiberis Rhizoma Recens]*  *Ziziphus jujuba Mill. [Rhamnaceae; Zizyphi Fructus]* | 33  17  17  28  5 |
| Fan  (2018) | Jianpijiedu Decoction | *Astragalus mongholicus Bunge [Fabaceae; Astragali Radix]*  *Erigeron breviscapus (Vaniot) Hand.-Mazz. [Asteraceae; Erigerontis Herba]*  *Agrimonia pilosa Ledeb. [Rosaceae; Agrimonia pilosa Ledebour]*  *Atractylodes lancea (Thunb.) DC. [Asteraceae; Atractylodis Rhizoma Alba]*  *Vitis vinifera L. [Vitaceae; Vitis vinifera]*  *Akebia trifoliata (Thunb.) Koidz. [Lardizabalaceae; Akebiae Fructus]*  *Coix lacryma-jobi var. ma-yuen (Rom.Caill.) Stapf [Poaceae; Coicis Semen]* | 14  14  14  11  19  9  19 |
| Bai  (2016) | Tongmai Sini Decoction | *Aconitum carmichaelii Debeaux [Ranunculaceae; Aconiti Lateralis Radix Preparata]*  *Glycyrrhiza glabra L. [Fabaceae; Glycyrrhizae Radix et Rhizoma]*  *Zingiber officinale Roscoe [Zingiberaceae; Zingiberis Rhizoma Recens]*  *Paeonia lactiflora Pall. [Paeoniaceae; Paeoniae Radix Alba]*  *Pheretima communissima [Lumbricidae; Lumbricus Corpus]*  *Codonopsis pilosula (Franch.) Nannf. [Campanulaceae; Codonopsis Pilosulae Radix]*  *Spatholobus suberectus Dunn [Fabaceae; Spatholobi Caulis]* | 22  11  20  14  9  9  15 |
| Lin  (2009) | Yiqi Huoxue Decoction | *Astragalus mongholicus Bunge [Fabaceae; Astragali Radix]*  *Atractylodes lancea (Thunb.) DC. [Asteraceae; Atractylodis Rhizoma Alba]*  *Coix lacryma-jobi var. ma-yuen (Rom.Caill.) Stapf [Poaceae; Coicis Semen]*  *Agrimonia pilosa Ledeb. [Rosaceae; Agrimonia pilosa Ledebour]*  *Salvia miltiorrhiza Bunge [Lamiaceae; Salviae Miltiorrhizae Radix]*  *Spatholobus suberectus Dunn [Fabaceae; Spatholobi Caulis]*  *Reynoutria japonica Houtt. [Polygonaceae; Polygoni Cuspidati Radix]* | No details |
| Liu  (2011) | HGWD | *Astragalus mongholicus Bunge [Fabaceae; Astragali Radix]*  *Neolitsea cassia (L.) Kosterm. [Lauraceae; Cinnamomi Ramulus]*  *Paeonia lactiflora Pall. [Paeoniaceae; Paeoniae Radix Alba]*  *Zingiber officinale Roscoe [Zingiberaceae; Zingiberis Rhizoma Recens]*  *Ziziphus jujuba Mill. [Rhamnaceae; Zizyphi Fructus]* | 36  14  18  14  18 |
| Tong  (2016) | Decoction for individual research | *Aconitum carmichaelii Debeaux [Ranunculaceae; Aconiti Lateralis Radix Preparata]*  *Angelica gigas Nakai [Apiaceae; Angelicae Gigantis Radix]*  *Dolomiaea costus (Falc.) Kasana & A.K.Pandey [Asteraceae; Aucklandiae Radix]*  *Syzygium aromaticum (L.) Merr. & L.M.Perry [Myrtaceae; Syzygii Flos]*  *Zingiber officinale Roscoe [Zingiberaceae; Zingiberis Rhizoma Recens]*  *Paeonia lactiflora Pall. [Paeoniaceae; Paeoniae Radix Rubra]*  *Neolitsea cassia (L.) Kosterm. [Lauraceae; Cinnamomi Ramulus]*  *Spatholobus suberectus Dunn [Fabaceae; Spatholobi Caulis]*  *Wolfiporia extensa [Polyporaceae; Poria Sclerotium]*  *Asarum heterotropoides F.Schmidt [Aristolochiaceae; Asiasari Radix et Rhizoma]* | 11  11  11  7  11  11  11  11  11  5 |
| Wang  (2016) | DGSNT | *Angelica gigas Nakai [Apiaceae; Angelicae Gigantis Radix]*  *Neolitsea cassia (L.) Kosterm. [Lauraceae; Cinnamomi Ramulus]*  *Paeonia lactiflora Pall. [Paeoniaceae; Paeoniae Radix Alba]*  *Asarum heterotropoides F.Schmidt [Aristolochiaceae; Asiasari Radix et Rhizoma]*  *Glycyrrhiza glabra L. [Fabaceae; Glycyrrhizae Radix et Rhizoma]*  *Tetrapanax papyrifer (Hook.) K.Koch [Araliaceae; Tetrapanacis Medulla]*  *Ziziphus jujuba Mill. [Rhamnaceae; Zizyphi Fructus]*  *Chaenomeles speciosa (Sweet) Nakai [Rosaceae; Chaenomelis Fructus]*  *Clematis terniflora var. mandshurica (Rupr.) Ohwi [Ranunculaceae; Clematidis Radix]*  *Tetradium ruticarpum (A.Juss.) T.G.Hartley [Rutaceae; Evodiae Fructus]*  *Astragalus mongholicus Bunge [Fabaceae; Astragali Radix]*  *Cucumis melo L. [Cucurbitaceae; Luffae Fructus Retinervus]* | 9  9  9  4  5  5  12  9  5  5  14  14 |
| Nishioka  (2011) | GJG | *Achyranthes bidentata Blume [Amaranthaceae; Achyranthis Radix]*  *Cornus officinalis Siebold & Zucc. [Cornaceae; Corni Fructus]*  *Wolfiporia extensa [Polyporaceae; Poria Sclerotium]*  *Paeonia × suffruticosa Andrews [Paeoniaceae; Moutan Radicis Cortex]*  *Dioscorea oppositifolia L. [Dioscoreaceae; Dioscoreae Rhizoma]*  *Plantago asiatica L. [Plantaginaceae; Plantaginis Semen]*  *Neolitsea cassia (L.) Kosterm. [Lauraceae; Cinnamomi Ramulus]*  *Rehmannia glutinosa (Gaertn.) Libosch. ex DC. [Orobanchaceae; Rehmanniae Radix Preparata]*  *Aconitum carmichaelii Debeaux [Ranunculaceae; Aconiti Lateralis Radix Preparata]*  *Alisma plantago-aquatica subsp. orientale (Sam.) Sam. [Alismataceae; Alismatis Rhizoma]* | 11  11  11  11  11  11  3  17  3  11 |
| Motoo  (2020) | Ninjin’yoeito | *Paeonia lactiflora Pall. [Paeoniaceae; Paeoniae Radix Alba]*  *Angelica gigas Nakai [Apiaceae; Angelicae Gigantis Radix]*  *Panax ginseng C.A.Mey. [Araliaceae; Ginseng Radix]*  *Atractylodes lancea (Thunb.) DC. [Asteraceae; Atractylodis Rhizoma Alba]*  *Astragalus mongholicus Bunge [Fabaceae; Astragali Radix]*  *Neolitsea cassia (L.) Kosterm. [Lauraceae; Cinnamomi Ramulus]*  *Citrus reticulata Blanco [Rutaceae; Citri Unshius Pericarpium]*  *Glycyrrhiza glabra L. [Fabaceae; Glycyrrhizae Radix et Rhizoma]*  *Rehmannia glutinosa (Gaertn.) Libosch. ex DC. [Orobanchaceae; Rehmanniae Radix Preparata]*  *Schisandra chinensis (Turcz.) Baill. [Schisandraceae; Schisandrae Fructus]*  *Senega officinalis Spach [Polygalaceae;Polygalae Radix]*  *Wolfiporia extensa [Polyporaceae; Poria Sclerotium]* | 6  13  9  12  6  9  6  5  12  3  6  13 |
| Li  (2006) | HGWD | *Astragalus mongholicus Bunge [Fabaceae; Astragali Radix]*  *Neolitsea cassia (L.) Kosterm. [Lauraceae; Cinnamomi Ramulus]*  *Angelica gigas Nakai [Apiaceae; Angelicae Gigantis Radix]*  *Wolfiporia extensa [Polyporaceae; Poria Sclerotium]*  *Paeonia lactiflora Pall. [Paeoniaceae; Paeoniae Radix Alba]*  *Spatholobus suberectus Dunn [Fabaceae; Spatholobi Caulis]*  *Sigesbeckia orientalis L. [Asteraceae; Siegesbeckiae Herba]*  *Paeonia lactiflora Pall. [Paeoniaceae; Paeoniae Radix Rubra]*  *Ziziphus jujuba Mill. [Rhamnaceae; Zizyphi Fructus]* | 13  8  10  10  13  13  13  13  7 |
| Bo  (2012) | Decoction for strengthen the spleen | *Panax ginseng C.A.Mey. [Araliaceae; Ginseng Radix]*  *Zingiber officinale Roscoe [Zingiberaceae; Zingiberis Rhizoma Recens]*  *Sinomenium acutum (Thunb.) Rehder & E.H.Wilson [Menispermaceae; Sinomeni Caulis et Rhizoma]*  *Adenophora triphylla (Thunb.) A.DC. [Campanulaceae; Adenophorae Radix]*  *Astragalus mongholicus Bunge [Fabaceae; Astragali Radix]*  *Atractylodes lancea (Thunb.) DC. [Asteraceae; Atractylodis Rhizoma Alba]*  *Paeonia lactiflora Pall. [Paeoniaceae; Paeoniae Radix Alba]*  *Glycyrrhiza glabra L. [Fabaceae; Glycyrrhizae Radix et Rhizoma]* | 11  11  16  30  11  7  7  7 |
| Jia  (2008) | Bu-yang-huan-wu-tang | *Astragalus mongholicus Bunge [Fabaceae; Astragali Radix]*  *Angelica gigas Nakai [Apiaceae; Angelicae Gigantis Radix]*  *Prunus persica (L.) Batsch [Rosaceae; Persicae Semen]*  *Paeonia lactiflora Pall. [Paeoniaceae; Paeoniae Radix Rubra]*  *Carthamus tinctorius L. [Asteraceae; Carthami Flos]*  *Conioselinum anthriscoides ‘Chuanxiong’ [Apiaceae; Cnidii Rhizoma]*  *Pheretima communissima [Lumbricidae; Lumbricus Corpus]*  *Spatholobus suberectus Dunn [Fabaceae; Spatholobi Caulis]*  *Curcuma longa L. [Zingiberaceae; Curcumae Radix]*  *Chaenomeles speciosa (Sweet) Nakai [Rosaceae; Chaenomelis Fructus]*  *Achyranthes bidentata Blume [Amaranthaceae; Achyranthis Radix]* | 21  10  7  7  7  7  7  10  7  7  10 |
| Liu  (2020) | Decoction for individual research | *Rehmannia glutinosa (Gaertn.) Libosch. ex DC. [Orobanchaceae; Rehmanniae Radix Preparata]*  *Cornus officinalis Siebold & Zucc. [Cornaceae; Corni Fructus]*  *Cullen corylifolium (L.) Medik. [Fabaceae; Psoraleae Semen]*  *Cuscuta chinensis Lam. [Convolvulaceae;Cuscutae Semen]*  *Angelica gigas Nakai [Apiaceae; Angelicae Gigantis Radix]*  *Conioselinum anthriscoides ‘Chuanxiong’ [Apiaceae; Cnidii Rhizoma]*  *Raphanus raphanistrum subsp. sativus (L.) Domin [Brassicaceae; Raphani Semen]*  *Paeonia lactiflora Pall. [Paeoniaceae; Paeoniae Radix Rubra]*  *Commiphora Commiphora myrrha (T.Nees) Engl. [Burseraceae; Myrrha] (T.Nees) Engl. [Burseraceae; Myrrha]*  *Salvia miltiorrhiza Bunge [Lamiaceae; Salviae Miltiorrhizae Radix]*  *Pheretima communissima [Lumbricidae; Lumbricus Corpus]*  *Bombyx mori L. [Bombycidae; Bombycis Corpus cum Batryticatus]*  *Citrus reticulata Blanco [Rutaceae; Citri Unshius Pericarpium]* | 18  12  8  7  7  6  8  6  3  7  6  6  6 |
| Tao  (2021) | Decoction for individual research | *Codonopsis pilosula (Franch.) Nannf. [Campanulaceae; Codonopsis Pilosulae Radix]*  *Wolfiporia extensa [Polyporaceae; Poria Sclerotium]*  *Atractylodes lancea (Thunb.) DC. [Asteraceae; Atractylodis Rhizoma Alba]*  *Glycyrrhiza glabra L. [Fabaceae; Glycyrrhizae Radix et Rhizoma]*  *Citrus reticulata Blanco [Rutaceae; Citri Unshius Pericarpium]*  *Pinellia ternata (Thunb.) Makino [Araceae; Pinelliae Tuber]*  *Dolomiaea costus (Falc.) Kasana & A.K.Pandey [Asteraceae; Aucklandiae Radix]*  *Wurfbainia villosa (Lour.) Škorničk. & A.D.Poulsen [Zingiberaceae; Amomi Fructus]*  *Inula japonica Thunb. [Asteraceae; Inulae Flos]*  *Philodendron insigne Schott [Araceae; Haematitum]*  *Ziziphus jujuba Mill. [Rhamnaceae; Zizyphi Fructus]*  *Chaenomeles speciosa (Sweet) Nakai [Rosaceae; Chaenomelis Fructus]*  *Astragalus mongholicus Bunge [Fabaceae; Astragali Radix]*  *Dioscorea oppositifolia L. [Dioscoreaceae; Dioscoreae Rhizoma]*  *Ligustrum lucidum W.T.Aiton [Oleaceae; Ligustri Lucidi Fructus]*  *Cuscuta chinensis Lam. [Convolvulaceae; Cuscutae Semen]*  *Scleromitrion diffusum (Willd.) R.J.Wang [Rubiaceae; Oldenlandiae Diffusae Herba]*  *Akebia trifoliata (Thunb.) Koidz. [Lardizabalaceae; Akebiae Fructus]*  *Polistes mandarinus Saussure et Geer [Vespidae; Vespae Nidus]*  *Paeonia × suffruticosa Andrews [Paeoniaceae; Moutan Radicis Cortex]*  *Glycyrrhiza glabra L. [Fabaceae; Glycyrrhizae Radix et Rhizoma]* | No details |
| Zu  (2023) | Decoction for individual research | *Codonopsis pilosula (Franch.) Nannf. [Campanulaceae; Codonopsis Pilosulae Radix]*  *Astragalus mongholicus Bunge [Fabaceae; Astragali Radix]*  *Dioscorea oppositifolia L. [Dioscoreaceae; Dioscoreae Rhizoma]*  *Coix lacryma-jobi var. ma-yuen (Rom.Caill.) Stapf [Poaceae; Coicis Semen]*  *Atractylodes lancea (Thunb.) DC. [Asteraceae; Atractylodis Rhizoma Alba]*  *Gallus gallus domesticus Briss. [Phasianidae; Galli Stomachichum Corium]*  *Hordeum vulgare L. [Poaceae; Hordei Fructus Germinatus]*  *Citrus medica L. var. sarcodactylis Swingle*  *Curcuma longa L. [Zingiberaceae; Curcumae Radix]*  *Bupleurum chinense DC. [Apiaceae; Bupleuri Radix]*  *Agrimonia pilosa Ledeb. [Rosaceae; Agrimonia pilosa Ledebour]*  *Scleromitrion diffusum (Willd.) R.J.Wang [Rubiaceae; Oldenlandiae Diffusae Herba]*  *Glycyrrhiza glabra L. [Fabaceae; Glycyrrhizae Radix et Rhizoma]* | 8  8  8  17  8  8  8  6  6  5  8  8  2 |
| Ho  (2022) | Yanghe Tongluo Decoction | *Rehmannia glutinosa (Gaertn.) Libosch. ex DC. [Orobanchaceae; Rehmanniae Radix Preparata]*  *Astragalus mongholicus Bunge [Fabaceae; Astragali Radix]*  *Cervus elaphus Linnaeus [Cervidae; Cervi Cornu]*  *Ephedra sinica Stapf [Ephedraceae; Ephedrae Herba]*  *Prunus persica (L.) Batsch [Rosaceae; Persicae Semen]*  *Angelica gigas Nakai [Apiaceae; Angelicae Gigantis Radix]*  *Carthamus tinctorius L. [Asteraceae; Carthami Flos]*  *Achyranthes bidentata Blume [Amaranthaceae; Achyranthis Radix]*  *Pheretima communissima [Lumbricidae; Lumbricus Corpus]*  *Terminalia chebula Retz. [Combretacea; Hirudo]*  *Neolitsea cassia (L.) Kosterm. [Lauraceae; Cinnamomi Ramulus]*  *Salvia miltiorrhiza Bunge [Lamiaceae; Salviae Miltiorrhizae Radix]*  *Paeonia lactiflora Pall. [Paeoniaceae; Paeoniae Radix Rubra]*  *Glycyrrhiza glabra L. [Fabaceae; Glycyrrhizae Radix et Rhizoma]*  *Brassica rapa L. [Brassicaceae; Brassicae Semen]*  *Zingiber officinale Roscoe [Zingiberaceae; Zingiberis Rhizoma Recens]* | 15  15  5  5  5  5  5  5  5  7  7  7  7  2  5 |
| Yu  (2024) | HGWD | *Astragalus mongholicus Bunge [Fabaceae; Astragali Radix]*  *Atractylodes lancea (Thunb.) DC. [Asteraceae; Atractylodis Rhizoma Alba]*  *Wolfiporia extensa [Polyporaceae; Poria Sclerotium]*  *Paeonia lactiflora Pall. [Paeoniaceae; Paeoniae Radix Alba]*  *Neolitsea cassia (L.) Kosterm. [Lauraceae; Cinnamomi Ramulus]*  *Salvia miltiorrhiza Bunge [Lamiaceae; Salviae Miltiorrhizae Radix]*  *Citrus reticulata Blanco [Rutaceae; Citri Unshius Pericarpium]*  *Liquidambar formosana Hance [Altingiaceae; Liquidambaris Fructus]*  *Angelica gigas Nakai [Apiaceae; Angelicae Gigantis Radix]*  *Ziziphus jujuba Mill. [Rhamnaceae; Zizyphi Fructus]*  *Zingiber officinale Roscoe [Zingiberaceae; Zingiberis Rhizoma Recens]*  *Glycyrrhiza glabra L. [Fabaceae; Glycyrrhizae Radix et Rhizoma]* | 28  7  7  7  7  7  7  7  7  7  6  3 |
| Lyu  (2021) | Decoction for individual research | *Astragalus mongholicus Bunge [Fabaceae; Astragali Radix]*  *Lonicera japonica Thunb. [Caprifoliaceae; Lonicerae Flos]*  *Angelica gigas Nakai [Apiaceae; Angelicae Gigantis Radix]*  *Glycyrrhiza glabra L. [Fabaceae; Glycyrrhizae Radix et Rhizoma]*  *Scolopendra subspinipes mutilans L. Koch [Scolopendridae; Scolopendra Corpus]*  *Neolitsea cassia (L.) Kosterm. [Lauraceae; Cinnamomi Ramulus]*  *Paeonia lactiflora Pall. [Paeoniaceae; Paeoniae Radix Rubra]*  *Paeonia lactiflora Pall. [Paeoniaceae; Paeoniae Radix Alba]*  *Clematis terniflora var. mandshurica (Rupr.) Ohwi [Ranunculaceae; Clematidis Radix]*  *Brassica rapa L. [Brassicaceae; Brassicae Semen]*  *Carthamus tinctorius L. [Asteraceae; Carthami Flos]* | 16  16  16  6  0.5  8  8  8  8  5.5  8 |

GJG, Gosha-jinki-gan; HGWD, Huangqi-Guizahi-Wuwu Decoction; DGSNT, Dang-Gui-Si-Ni-Tang

# S4. Supplementary Table 2

#### Table 2. A priori algorithm-based association rules for THM prescribed for the prevention of CIPN.

| No. | Association Rules | Support (%) | Confidence (%) | Lift* |
| --- | --- | --- | --- | --- |
| 1 | *Angelica gigas Nakai*  *[Apiaceae; Angelicae Gigantis Radix]* (當歸)  *→ Paeonia lactiflora Pall.*  *[Paeoniaceae; Paeoniae Radix Rubra]* (赤芍藥) | 23.684 | 100.0 | 2.235 |
| 2 | *Neolitsea cassia (L.) Kosterm.*  *[Lauraceae; Cinnamomi Ramulus]* (桂枝)  *→ Paeonia lactiflora Pall.*  *[Paeoniaceae; Paeoniae Radix Alba]* (白芍藥)*,*  *Angelica gigas Nakai*  *[Apiaceae; Angelicae Gigantis Radix]* (當歸) | 23.684 | 100.0 | 1.583 |
| 3 | *Neolitsea cassia (L.) Kosterm.*  *[Lauraceae; Cinnamomi Ramulus]* (桂枝)  *→ Rehmannia glutinosa (Gaertn.) Libosch. ex DC.*  *[Orobanchaceae; Rehmanniae Radix Preparata]*  (熟地黃) | 21.053 | 87.5 | 1.385 |
| 4 | *Neolitsea cassia (L.) Kosterm.*  *[Lauraceae; Cinnamomi Ramulus]* (桂枝)  *→ Achyranthis Radix, Achyranthes bidentata Blume*  *[Amaranthaceae; Achyranthis Radix]* (牛膝) | 18.421 | 85.714 | 1.357 |
| 5 | *Angelica gigas Nakai*  *[Apiaceae; Angelicae Gigantis Radix]* (當歸)  *→ Paeonia lactiflora Pall.*  *[Paeoniaceae; Paeoniae Radix Rubra]* (赤芍藥),  *Astragali Radix, Astragalus mongholicus Bunge [Fabaceae; Astragali Radix]* (黃芪) | 18.421 | 100.0 | 2.235 |
| 6 | *Neolitsea cassia (L.) Kosterm.*  *[Lauraceae; Cinnamomi Ramulus]* (桂枝)  *→ Paeonia lactiflora Pall.*  *[Paeoniaceae; Paeoniae Radix Alba]* (白芍藥),  *Angelica gigas Nakai*  *[Apiaceae; Angelicae Gigantis Radix]* (當歸),  *Astragali Radix, Astragalus mongholicus Bunge [Fabaceae; Astragali Radix]* (黃芪) | 18.421 | 100.0 | 1.583 |
| 7 | *Angelica gigas Nakai*  *[Apiaceae; Angelicae Gigantis Radix]* (當歸)  *→ Conioselinum anthriscoides ‘Chuanxiong’*  *[Apiaceae; Cnidii Rhizoma]]* (川芎) | 15.789 | 100.0 | 2.235 |
| 8 | *Astragali Radix, Astragalus mongholicus Bunge [Fabaceae; Astragali Radix]* (黃芪)  *→ Carthamus tinctorius L.*  *[Asteraceae; Carthami Flos]* (紅花) | 15.789 | 100.0 | 1.407 |
| 9 | *Angelica gigas Nakai*  *[Apiaceae; Angelicae Gigantis Radix]* (當歸)  *→ Paeonia lactiflora Pall.*  *[Paeoniaceae; Paeoniae Radix Rubra]* (赤芍藥),  *Neolitsea cassia (L.) Kosterm.*  *[Lauraceae; Cinnamomi Ramulus]* (桂枝) | 15.789 | 100.0 | 2.235 |

* Value of minimum antecedent support: 15.0%; minimum rule confidence: 85.0%; maximum number of antecedents: 5. Listed in descending order of Support

CIPN, chemotherapy-induced peripheral neuropathy; THM, traditional herbal medicine.
